# Early differential responses elicited by BRAF^V600E^ in adult mouse models

**DOI:** 10.1038/s41419-022-04597-z

**Published:** 2022-02-10

**Authors:** Giuseppe Bosso, Pablo Lanuza-Gracia, Sergio Piñeiro-Hermida, Merve Yilmaz, Rosa Serrano, Maria A. Blasco

**Affiliations:** Telomeres and Telomerase Group, Molecular Oncology Program, Spanish National Cancer Centre (CNIO), Melchor Fernández Almagro 3, Madrid, E-28029 Spain

**Keywords:** Lung cancer, Senescence

## Abstract

The BRAF gene is frequently mutated in cancer. The most common genetic mutation is a single nucleotide transition which gives rise to a constitutively active BRAF kinase (BRAF^V600E^) which in turn sustains continuous cell proliferation. The study of BRAF^V600E^ murine models has been mainly focused on the role of BRAF^V600E^ in tumor development but little is known on the early molecular impact of BRAF^V600E^ expression in vivo. Here, we study the immediate effects of acute ubiquitous BRAF^V600E^ activation in vivo. We find that BRAF^V600E^ elicits a rapid DNA damage response in the liver, spleen, lungs but not in thyroids. This DNA damage response does not occur at telomeres and is accompanied by activation of the senescence marker p21^CIP1^ only in lungs but not in liver or spleen. Moreover, in lungs, BRAF^V600E^ provokes an acute inflammatory state with a tissue-specific recruitment of neutrophils in the alveolar parenchyma and macrophages in bronchi/bronchioles, as well as bronchial/bronchiolar epithelium transdifferentiation and development of adenomas. Furthermore, whereas in non-tumor alveolar type II (ATIIs) pneumocytes, acute BRAF^V600E^ induction elicits rapid p53-independent p21^CIP1^ activation, adenoma ATIIs express p53 without resulting in p21^CIP1^ gene activation. Conversely, albeit in Club cells BRAF^V600E^-mediated proliferative cue is more exacerbated compared to that occurring in ATIIs, such oncogenic stimulus culminates with p21^CIP1^-mediated cell cycle arrest and apoptosis. Our findings indicate that acute BRAF^V600E^ expression drives an immediate induction of DNA damage response in vivo. More importantly, it also results in rapid differential responses of cell cycle and senescence-associated proteins in lung epithelia, thus revealing the early molecular changes emerging in BRAF^V600E^-challenged cells during tumorigenesis in vivo.

## Introduction

The BRAF gene, encoding a master kinase of RAS-activated RAF-MEK-ERK (RAS-) pathway, is frequently mutated in human malignancies [1–6]. More than 90% of these mutations affect codon 600 of the BRAF protein, and out of these, ~90% represent the 1799T > A nucleotide transition, which results in a constitutively active [7] BRAF variant (BRAF^V600E^) which indefinitely sustains cell proliferation.

The investigation of BRAF^V600E^ genetically engineered mouse models (GEMMs) has been focused on the role of BRAF^V600E^ in cancer in diverse tissues/organs [8–15]. BRAF^V600E^ expression in vivo triggers an early hyperplastic growth which culminates in a proliferative arrest known as oncogene-induced senescence (OIS) [9, 11, 15], which is driven by p53/p21^CIP1^ and retinoblastoma protein (Rb)/p16^INK4a^ pathways [16, 17].

Albeit the above-mentioned GEMMs, where BRAF^V600E^ expression relies on tissue-specific promoters, allowed to dissect the function of BRAF^V600E^ in cancer, such an approach carries the limitation of lacking the global view of potential early effects induced by this oncogene. Indeed, apart from the initial wave of hyperplasia, the instant consequences on BRAF^V600E^-activation in vivo remain unexplored. Here we study the early events following acute expression of BRAF^V600E^ in vivo.

## Results

### Ubiquitous acute conditional activation of BRAF^V600E^ allele is lethal in adult mice

To analyze the immediate impact of ubiquitous expression of BRAF^V600E^ in vivo, we generated UbiCreER^T2/+^;BRAF^LSL_V600E/+^ (BRAF^V600E^) mice harboring UbiCreER^T2^ allele [18], expressing the conditionally active CreER^T2^ recombinase gene under the control of the human ubiquitin promoter (UbiCreER^T2^), combined with BRAF^LSL_V600E^ allele [9]. First, we checked the viability of BRAF^V600E^ mice in the absence of tamoxifen treatments. Albeit until the age of 9 weeks all the mice appeared healthy, starting from 10 weeks from birth they showed weight loss, locomotion alteration, bad shape, papillomatous skin lesions and all of them died between 12–18 weeks from birth (Supplementary Fig. 1A, B), a phenotype which is most likely due to the effects of a spontaneous Cre-mediated recombination of BRAF^V600E^ allele over time. Consistently, PCR analysis of spontaneous papilloma-like lesions arisen in some of the tamoxifen-untreated BRAF^V600E^ mice revealed Cre-mediated activation of BRAF^V600E^ allele (Supplementary Fig. 1C).

To induce an acute BRAF^V600E^ activation, 7–8 weeks old mice were administered tamoxifen intraperitoneally. The BRAF^V600E^ mice, but not the UbiCreER^T2/+^ (control) strain, started to appear sick 2–3 days post-injection and needed to be euthanatized after 3–5 days (Supplementary Fig. 2A, B). Macroscopic analysis revealed that BRAF^V600E^ mice had pale livers, which may be indicative of hepatic steatosis (HS) (Supplementary Fig. 2C). PCR analysis confirmed Cre-mediated rearrangement of BRAF^V600E^ allele upon tamoxifen administration, resulting in BRAF^V600E^ expression in all the tissues/organs analyzed (Supplementary Fig. 2D).

### Early effects of BRAF^V600E^ expression in thyroids, liver and spleen

First, we analyzed the early effects of BRAF^V600E^ expression in thyroids. Hematoxylin eosin (H&E) staining revealed no morphological alterations between BRAF^V600E^ and control thyroids at 4–5 days after Cre-induction (Supplementary Fig. 2E). Phosphorylation analysis of the downstream effector ERK kinase (ppERK) revealed an increase in ppERK-positive cells in BRAF^V600E^ thyroids compared to control, thus confirming that BRAF^V600E^ is induced in thyroid glands and is stimulating the RAS-pathway (Supplementary Fig. 2F). However, BRAF^V600E^ thyroids displayed no changes in apoptosis, as determined by caspase 3 (CC3), in senescence as determined by p21^CIP1^, or in DNA damage as indicated by the DNA damage protein γH2AX (Supplementary Fig. 2G–I) compared to controls, indicating that acute BRAF^V600E^ expression in thyroids does not have an apparent impact on cellular viability programs.

**Fig. 1:**
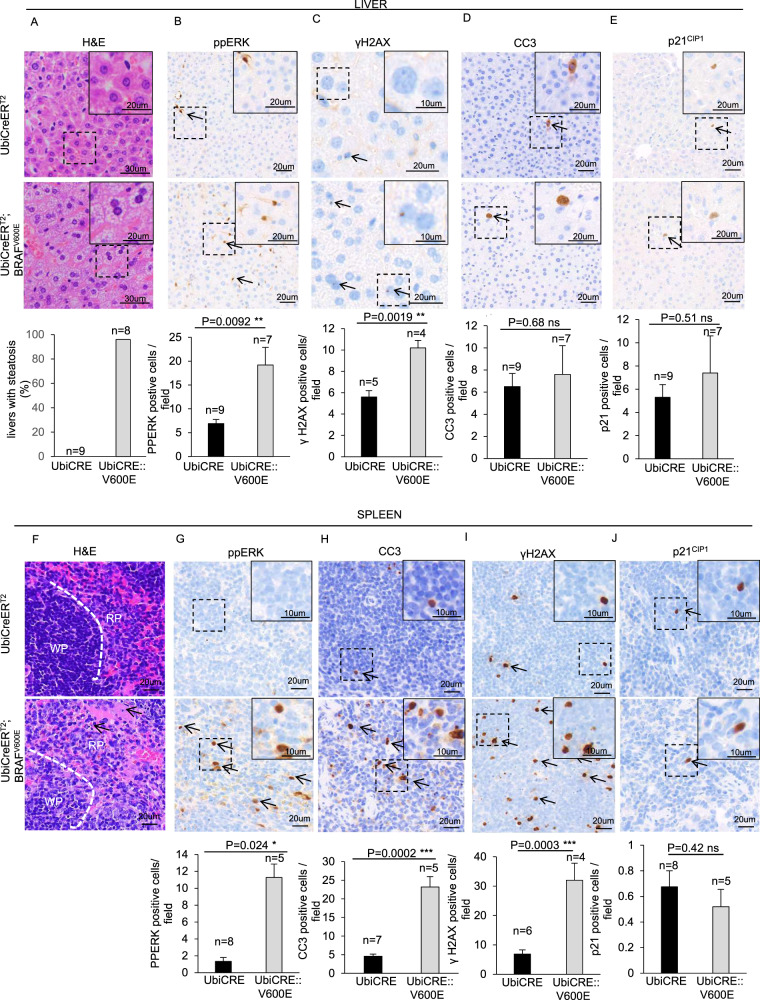
Early effects of ubiquitous expression of BRAF^V600E^ in liver and spleen. **A**–**E** Representative images and quantifications showing **A** H&E staining, **B** ppERK, **C** γH2AX, **D** CC3 and **E** p21^CIP1^ immunostainings in liver sections from BRAF^V600E^ and control mice. **F**–**J** Representative images and quantifications showing **F** H&E staining, **G** PPERK, **H** CC3, **I** γH2AX and **J** p21^CIP1^ immunostainings in spleen sections from BRAF^V600E^ and control mice. Quantifications were performed on five different areas of the sections in a random way. Data are expressed as mean ± SEM; *n* = animals per group. *P < 0.05; **P < 0.01; ***P < 0.001, ns = not significant. (T Student’s test unpaired). Arrows point to selected positive cells for the indicated marker. Legend for figure F: WP = White Pulp, RD = Red Pulp; the white dashed line marks the boundary between white and red pulps. The arrows point to apoptotic bodies. Insets: magnifications of areas inside dashed squares.

Next, we checked the immediate effects of BRAF^V600E^ expression in the liver. In agreement with pale livers present in BRAF^V600E^ mice at their end-point, H&E staining revealed the presence of microvesicular HS which was not present in age-matched controls (Fig. 1A) (see Discussion). BRAF^V600E^ livers showed an enrichment in ppERK-positive cells, thus confirming RAS-pathway activation (Fig. 1B). Concomitantly, albeit γH2AX-positive BRAF^V600E^ hepatocytes were increased 2-fold compared to controls, neither CC3- nor p21^CIP1^-positive cells were significantly altered (Fig. 1C–E), thus suggesting that after 3–5 days of acute expression, although BRAF^V600E^ induces HS and DNA damage, it elicits neither apoptosis nor alterations in p21^CIP1^ expression in the liver.

We next investigated the effects of BRAF^V600E^ activation in the spleen. Histopathology analyses revealed a substantial loss of cellularity in both white and red pulp zones as well as the appearance of apoptotic bodies in BRAF^V600E^ spleens but not in controls (Fig. 1F). Only BRAF^V600E^ spleens displayed loss of a clear boundary between the white and red pulp (Fig. 1F), which is indicative of a general cell depletion [19]. Although we found ppERK activation in BRAF^V600E^ spleens (Fig. 1G), Ki67-positive proliferating BRAF^V600E^ cells were significantly reduced (Supplementary Fig. 3), which is in agreement with the observed loss of cellularity. Coherently, CC3-positive cells were increased compared to controls, suggesting that acute BRAF^V600E^ expression in the spleen results in lower proliferation and apoptosis induction (Fig. 1H). Interestingly, γH2AX-positive cells in BRAF^V600E^ spleens were increased by 7-fold compared to controls suggestive of enhanced DNA damage following BRAF^V600E^ expression (Fig. 1I). In contrast, we observed no accumulation of p21^CIP1^ [20] (Fig. 1J). Collectively, in the spleen acute BRAF^V600E^ induction induces DNA damage without eliciting p21^CIP1^ expression.

### Early effects of BRAF^V600E^ expression in lung alveolar parenchyma

**Fig. 2:**
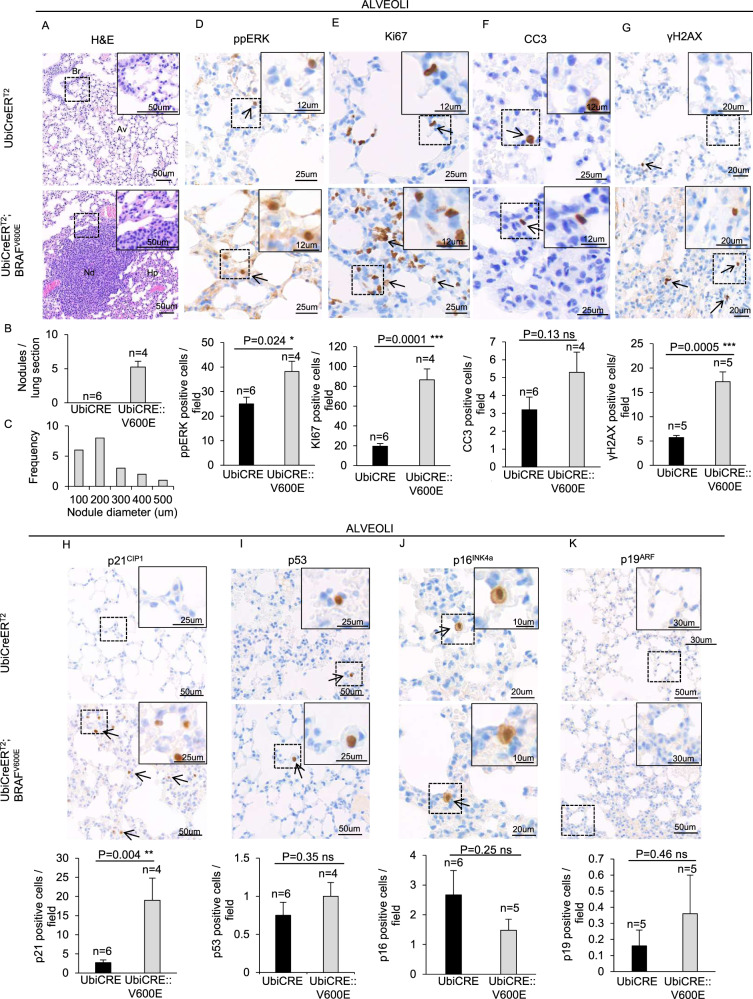
Early effects of BRAF^V600E^ expression in the alveolar parenchyma. Representative images and quantifications showing **A**–**C** H&E staining, **D** ppERK, **E** Ki67, **F** CC3, **G** γH2AX, **H** p21^CIP1^, **I** p53, **J** p16^INK4a^, **K** p19^ARF^ immunostainings in lung sections from BRAF^V600E^ and control mice. Quantifications were performed on five different areas of the sections in a random way. Data are expressed as mean ± SEM; *n* = animals per group. *P < 0.05; **P < 0.01; ***P < 0.001, ns = not significant. (T Student’s test unpaired). Arrows point to selected positive cells for the indicated marker. Legend for figure **A**: Br: bronchus, Av: Alveolus, Nd: hyperplastic nodule, Hp: hyperplastic alveolar epithelium. Legend for **I**: the arrows point to neutrophils. Figures **B** and **C** represent the quantifications of the nodules shown in **A**. Insets: magnifications of areas inside dashed squares.

To investigate the immediate effects of BRAF^V600E^ expression in lungs, we first confirmed that BRAF^V600E^ was indeed expressed in lungs at the protein level by immunoblot analysis (Supplementary Fig. 4). Next, we found that BRAF^V600E^ alveolar parenchyma showed alveolar wall thickening and adenomas (Fig. 2A–C). An increase in ppERK- and Ki67-positive cells confirmed that BRAF^V600E^ leads to RAS-pathway activation and consequent proliferation in lungs (Fig. 2D, E). We found no differences in alveolar CC3-positive cells between BRAF^V600E^ and control mice (Fig. 2F). Interestingly, BRAF^V600E^ expression elicited a significant increment in γH2AX and p21^CIP1^ protein levels, as well as in the number cells positive for such markers (Supplementary Fig. 4, Fig. 2G, H). Moreover, telomere-induced foci (TIF) analysis revealed that BRAF^V600E^-elicited DNA damage is not telomeric (Supplementary Fig. 5A). However, we found no changes in the frequency of cells expressing either p53, or p16^INK4a^ and p19^ARF^, which are all expressed in senescent BRAF^V600E^-driven lung adenomas [21] (Fig. 2I–K).

Altogether, these data suggest that in the alveolar parenchyma BRAF^V600E^ elicits rapid DNA damage and p53-independent p21^CIP1^ activation without inducing other classical hallmarks of senescence.

### Effects of BRAF^V600E^ expression in lung adenomas

**Fig. 3:**
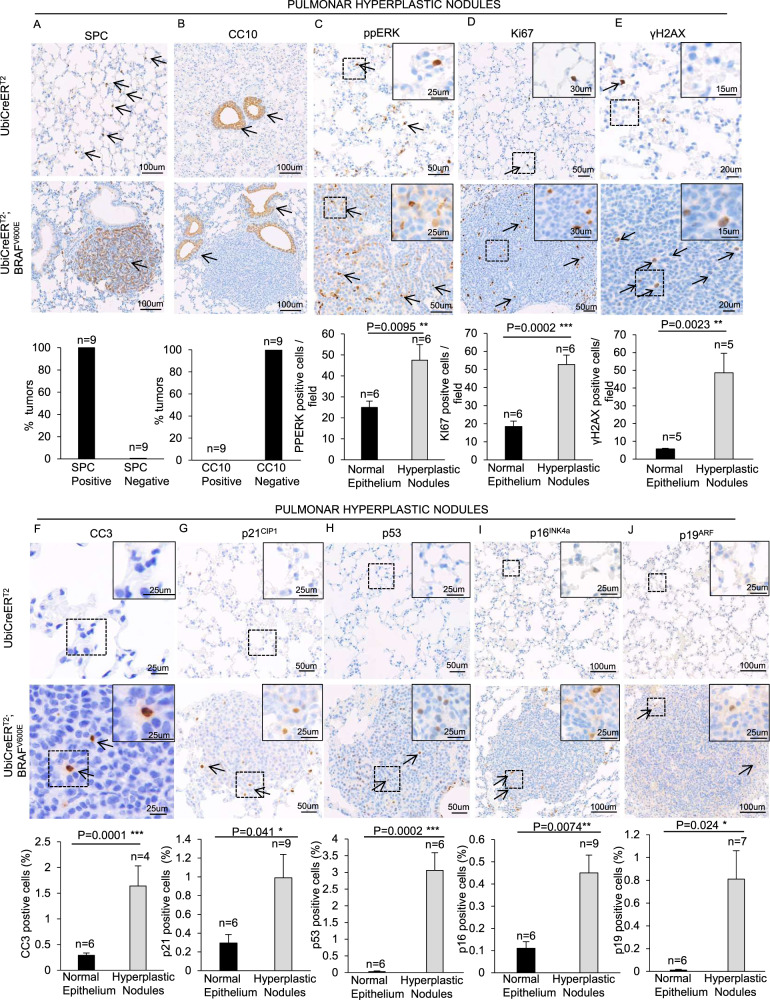
Effects of expression of BRAF^V600E^ in hyperplastic nodules. Representative images (top) and quantifications (bottom) showing H&E staining, **A** SPC, **B** CC10, **C** ppERK, **D** Ki67, **E** γH2AX, **F** CC3, **G** p21^CIP1^, **H** p53, **I** p16^INK4a^, **J** p19^ARF^ immunostainings in lung sections from BRAF^V600E^ and control mice. Quantifications were performed on four different random areas of at least four hyperplastic nodules sections and four different random areas of uninduced alveolar parenchyma. Data are expressed as mean ± SEM; *n* represents respectively the number of animals per group in “normal epithelium” samples and the number of tumors in the “hyperplastic nodules” samples. For each condition at least 4 mice were used. *P < 0.05; **P < 0.01; ***P < 0.001, ns = not significant (T Student’s test unpaired). Arrows point to selected positive cells for the indicated marker. Insets: magnifications of areas inside dashed squares.

The finding that BRAF^V600E^ mice displayed adenomas (Fig. 2A–C) staining positive for the prosurfactant protein C (SPC), a marker of alveolar type II cells (ATIIs), and negative for CC10, a protein specifically expressed by Club cells (CCs), confirmed the evidence that BRAF^V600E^-induced adenomas display the properties of ATIIs [9, 17] (Fig. 3A, B). Although the adenomas showed high levels of ppERK and Ki67, which are indicative of the BRAF^V600E^-triggered proliferative wave (Fig. 3C, D), they also showed increased DNA damage compared to controls (Fig. 3E). Notably, as occurred in normal alveoli, even in the adenomas, BRAF^V600E^-induced DNA damage is not telomeric (Supplementary Fig. 5B). Tumors also showed increased expression of CC3 and p21^CIP1^, whereas p53^+^ cells were incremented by 500-fold compared to control, thus suggesting apoptosis, p21 and p53 induction in the adenomas (Fig. 3F–H). Moreover, we detected enriched protein levels of the senescent markers p15^INK4b^, p16^INK4a^ and p27^KIP1^ (Supplementary Fig. 4), as well as in the occurrence of p16^INK4a^- and p19^ARF^-positive cells (Fig. 3I, J), therefore indicating that senescence is taking place in some of the adenoma cells. Collectively, these findings indicate that BRAF^V600E^-driven adenomas are composed by both proliferating and senescent cells.

Albeit in BRAF^V600E^ mice both non-tumor alveolar parenchyma and adenomas showed increased p21^CIP1^ levels compared to controls (Fig. 2H, Fig. 3G, Supplementary Fig. 4), neither smaller nor larger adenomas show significant changes in p21^CIP1^ levels compared to non-tumor BRAF^V600E^ alveolar zones (Supplementary Fig. 6A). Nevertheless, only adenomas showed p53, p16^INK4a^ and p19^ARF^ upregulation (Fig. 2I–K, Fig. 3H–J). Thus, we hypothesized that p21^CIP1^ may be activated *via* p53 only in adenomas and that alternative mechanisms might be employed to induce p21^CIP1^ in BRAF^V600E^-challenged non-tumor alveoli which might not reflect the activation of classical OIS.

BRAF^V600E^ is known to activate p21^CIP1^ expression *via* E2F transcription factors in vitro, upon cyclin-dependent kinases (CDKs)-mediated Rb inactivation [22, 23]. Nevertheless, no alterations in the percentage of cells showing the inactivated phosphorylated version of Rb was observed either between BRAF^V600E^ non-tumor areas and adenomas or between BRAF^V600E^ and control mice (Supplementary Fig. 6B), thus suggesting that BRAF^V600E^-mediated p21^CIP1^ activation in the alveolar parenchyma may not rely on pRb/E2F axis.

The RAS-pathway also induces small mother against decapentaplegic-3 (SMAD3) [24, 25] and STAT3 [26, 27], two transcriptions factors whose phosphorylated versions (pSMAD3, pSTAT3) activate p21^CIP1^ gene [28–31]. Unexpectedly, we found no changes in the percentage of pSMAD3-positive cells among adenomas and non-tumor alveolar areas from both BRAF^V600E^ mice and controls (Supplementary Fig. 6C). Conversely, both non-tumor BRAF^V600E^ parenchyma and adenomas showed increased pSTAT3 staining compared to controls (Supplementary Fig. 6D), thus indicating that BRAF^V600E^ activates STAT3, but not SMAD3, in the lung and potentially suggesting that p21^CIP1^ may be induced *via* STAT3 in BRAF^V600E^ lung parenchyma.

### Molecular differences between BRAF^V600E^-challenged tumor and non-tumor ATIIs

**Fig. 4:**
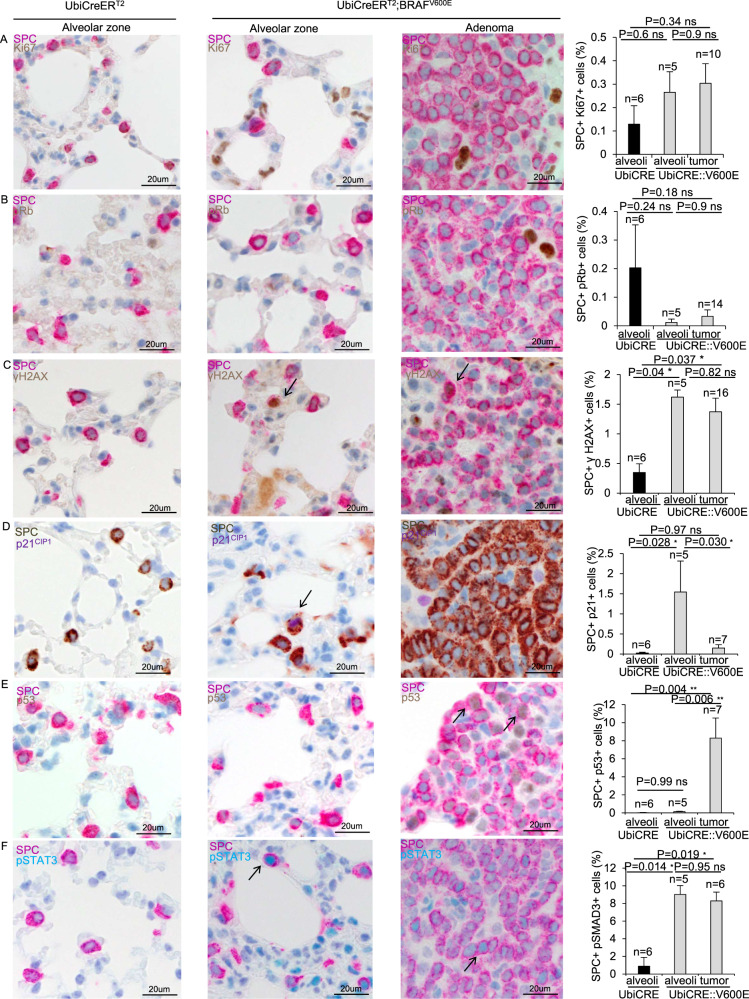
BRAF^V600E^ induction results in differential expression of cell cycle and senescence markers in ATII cells from uninduced, induced non-tumor alveolar parenchyma and lung adenomas. Representative images (from the left) and quantifications (right) showing SPC+ cells staining positive for **A** Ki67, **B** pRb, **C** γH2AX, **D** p21^CIP1^, **E** p53 and **F** pSTAT3 double immunostainings in normal alveolar epithelium of control mice (left), non-tumor areas (center) and adenomas (right) from lung sections of BRAF^V600E^ mice. Quantifications were performed on four to ten different random areas of at least four hyperplastic nodules sections and four to ten different random areas of uninduced alveolar parenchyma. Data are expressed as mean ± SEM; *n* represents respectively the number of animals per group in “alveoli” samples and the number of adenomas in the “tumor” samples. For each condition at least 4 mice were used. The percentages shown in the charts were obtained dividing the number of double positive cells by the overall number of SPC + cells. *P < 0.05; **P < 0.01; ***P < 0.001, ns = not significant. (ANOVA test with Tukey’s post-hoc correction). Arrows point to selected positive cells for the indicated marker.

To better analyze the differential response elicited by BRAF^V600E^ in ATIIs, we checked whether BRAF^V600E^ might affect the proliferation index of non-tumor and adenoma ATIIs. Double immunohistochemistry stainings with SPC and Ki67 markers revealed that upon BRAF^V600E^ activation, albeit both non-tumor and tumor ATIIs from BRAF^V600E^ mice tended to display a higher proliferation index, we found no significant changes in the number of SPC^+^Ki67^+^ cells (Fig. 4A). Consistently, the percentage of ATIIs expressing pRb, another hallmark of cell cycle progression, among adenomas and non-tumor alveolar areas from both BRAF^V600E^ and control mice was not affected (Fig. 4B), therefore enforcing the evidence that acute BRAF^V600E^ activation does not result in drastic changes in the expression of proliferative markers in ATIIs. Interestingly, both non-tumor and tumor BRAF^V600E^ ATIIs displayed increased DNA damage (Fig. 4C). Remarkably, only non-tumor BRAF^V600E^ ATIIs showed an increment in p21^CIP1^ expression compared to both controls and adenoma ATIIs (Fig. 4D), thus indicating that the majority of p21^CIP1^-positive cells in adenomas are not SPC^+^ATIIs and that the oncogenic challenge elicits a rapid p21^CIP1^activation in ATIIs before they can give rise to adenomas (Supplementary Fig. 6A). Surprisingly, whereas there was no alteration in p53 expression in non-tumor BRAF^V600E^ ATIIs, we observed a robust p53 induction in adenomas (Fig. 4E), thus suggesting that p21^CIP1^ activation in non-tumor ATIIs does not rely on p53 and that p53 induction in adenoma ATIIs does not promptly result in p21^CIP1^ expression. Moreover, double staining experiments by using SPC marker combined with either pSMAD3 (Supplementary Fig. 7) or pSTAT3 (Fig. 4F) showed a drastic increment in pSTAT3, but not in pSMAD3, in BRAF^V600E^ mice compared to control, thus confirming that 1) the BRAF^V600E^-dependent increase of pSTAT3 in the lungs (Supplementary Fig. 6D) is ascribable to pSTAT3 enrichment in ATIIs and 2) arguing for the possibility that pSTAT3 activation may be uncoupled from cell proliferation in BRAF^V600E^-challenged ATIIs, at least at the specific stages of oncogene-induced cell transformation analyzed (Fig. 4A, B, F).

Altogether, these findings indicate that albeit BRAF^V600E^ induces DNA damage as well as pSTAT3 in ATIIs outside and inside the adenomas, it results in differential p21^CIP1^ and p53 expression in non-tumor and adenoma ATIIs.

### Early effects of BRAF^V600E^ in bronchial/bronchiolar epithelium and Club cells

**Fig. 5:**
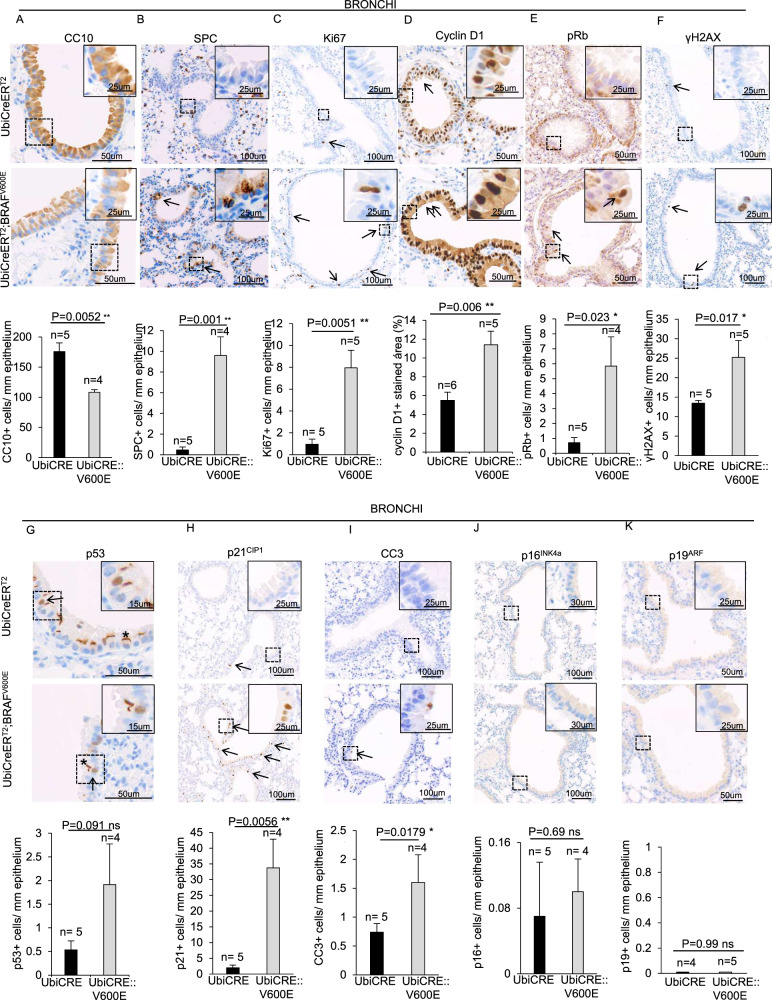
Analysis of the early molecular response of BRAF^V600E^ induction in bronchial/bronchiolar epithelium. **A**–**K** Representative images (top) and quantifications (bottom) showing bronchial/bronchiolar epithelial cells staining positive for **A** CC10, **B** SPC, **C** Ki67, **D** cyclin D1, **E** pRb, **F** γH2AX, **G** p53, **H** p21^CIP1^, **I** CC3, **J** p16^INK4a^, **K** p19^ARF^ immunostainings in lung sections from BRAF^V600E^ and control mice. Quantifications were performed on at least five different areas of the lung sections in a random way. Data are expressed as mean ± SEM; *n* = animals per group. *P < 0.05; **P < 0.01; ***P < 0.001, ns = not significant. (T Student’s test unpaired). Arrows point to selected positive cells for the indicated marker. Insets: magnifications of areas inside dashed squares. Asterisks in **G** indicate an unspecific signal of the antibody.

We next studied the early effects of BRAF^V600E^ expression in bronchi/bronchioles. Following BRAF^V600E^ activation, bronchial/bronchiolar cells showed a significant loss of cells positive for the specific CC marker CC10 (Club cell secretory protein 10KDa) (Fig. 5A). Furthermore, intensity of CC10 staining was also significantly reduced (Supplementary Fig. 8A). Concomitantly, SPC^+^ intrabronchial cells were drastically increased (Fig. 5B), thus suggesting that BRAF^V600E^ induces transdifferentiation of CCs into ATIIs. Moreover, an increment in Ki67-positive cells (Fig. 5C) was accompanied by respectively a 2-fold and 8-fold increase in the intensity of cyclin D1 staining and in the number of pRb-positive cells in BRAF^V600E^ bronchi/bronchioles compared to controls (Fig. 5D, E), therefore indicating a dramatic stimulation of cell cycle progression. Simultaneously, BRAF^V600E^ mice displayed a 2-fold increase of γH2AX-positive cells (Fig. 5F) and although they tended to display more p53^+^ cells, such an increase did not reach statistical significance. Nevertheless, p21^CIP1^- and CC3-positive bronchial/bronchiolar BRAF^V600E^ cells were enriched by 15-fold and 2-fold respectively compared to controls (Fig. 5H, I), thus suggesting that the dramatic proliferative cues observed upon BRAF^V600E^ challenge culminates in a robust p53-independent p21-mediated cell cycle arrest and cell death. Intriguingly, there were no changes in the frequency of p16^INK4a^- and p19^ARF^-positive cells (Fig. 5J, K), thus suggesting that p21^CIP1^ increase is not coupled to other bronchi/bronchioles senescence markers [32].

**Fig. 6:**
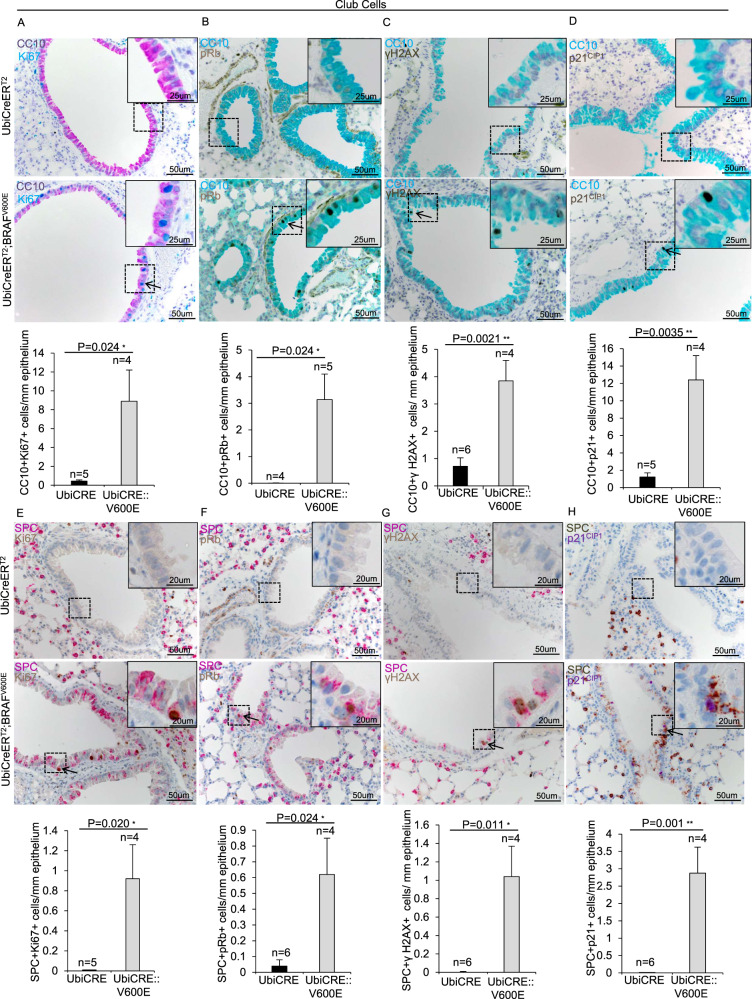
Analysis of the early molecular response of BRAF^V600E^ induction in Club cells. **A**–**D** Representative images (top) and quantifications (bottom) showing CC10+ cells staining positive for **A** Ki67, **B** pRb, **C** γH2AX, **D** p21^CIP1^ double immunostainings in lung sections from BRAF^V600E^ and control mice. **E**–**H** Representative images (top) and quantifications (bottom) showing bronchial/bronchiolar SPC + cells staining positive for **E** Ki67, **F** pRb, **G** γH2AX, **H** p21^CIP1^ double immunostainings in lung sections from BRAF^V600E^ and control mice. Quantifications were performed on at least five different areas of the lung sections in a random way. Data are expressed as mean ± SEM; *n* = animals per group. *P < 0.05; **P < 0.01; ***P < 0.001, ns = not significant. (T Student’s test unpaired). Arrows point to selected positive cells for the indicated marker. Insets: magnifications of areas inside dashed squares.

To investigate the early BRAF^V600E^-driven responses in CCs, we first checked the proliferation index of BRAF^V600E^ CCs. Contrary to ATIIs, double immunohistochemistry staining with CC10 and Ki67 markers revealed that BRAF^V600E^ CCs showed a dramatic 20-fold and 15-fold increase respectively in Ki67 and pRb (Fig. 6A, B), thus confirming the finding that CCs are sensitive to BRAF^V600E^-mediated proliferation stimulation. However, additional double immunostainings with CC10, γH2AX and p21^CIP1^ markers revealed that BRAF^V600E^ CCs showed respectively a 5.4-fold and 10-fold increase in γH2AX- and p21-positive cells compared to controls (Fig. 6C, D), therefore enforcing the evidence that BRAF^V600E^ also elicits a dramatic induction of DNA damage and p21^CIP1^ in CCs.

Interestingly, we found the same expression pattern described above also in SPC^+^CCs. Indeed, transdifferentiating BRAF^V600E^ CCs were positive for Ki67 and pRb (Fig. 6E, F), as well as for γH2AX and p21^CIP1^ markers (Fig. 6G, H), thus indicating that the BRAF^V600E^-mediated proliferation stimulation coexists with the cytotoxic response during the early steps of CC-to-ATII transdifferentiation. It is worth pointing out that bronchial/bronchiolar cells staining positive or negative for SPC show the same frequency in p21^CIP1^-, γH2AX-, Ki67-, pRb-positive cells (Supplementary Fig. 8B–E), thus suggesting that the increment respectively in p21^CIP1^ expression, DNA damage and proliferation may not affect the onset of the transdifferentiation process.

Altogether, these findings unveil that upon BRAF^V600E^-challenge CCs transdifferentiate and massively activate a robust cell cycle progression signaling which rapidly culminates in cell cycle inhibition and apoptosis.

### p21^CIP1^ activation in lungs is the consequence of acute tamoxifen-mediated BRAF^V600E^ induction

Next, we ruled out the possibility that p53-independent p21^CIP1^ activation in bronchi/bronchioles and in non-tumor alveolar parenchyma might be ascribable to a prolonged effect of BRAF^V600E^ chronic activation, which may be spontaneously occurred at some earlier time-points before tamoxifen injections. For this purpose, concomitantly with tamoxifen-treated BRAF^V600E^ mice, we also analyzed the lungs of 10–11 weeks old BRAF^V600E^ mice without previous tamoxifen treatment. Remarkably, although such untreated mice showed spontaneous lung adenomas expressing high levels of p21^CIP1^ and p53, we found no differences in both these proteins in non-tumor alveolar parenchyma of BRAF^V600E^ mice compared to untreated age-matched controls (Supplementary Fig. 9A, B). Similarly, we observed no change in either Ki67 or p21^CIP1^ in bronchi/bronchioles compared to controls (Supplementary Fig. 9C, D), thus enforcing the evidence that the proliferation induction and p21^CIP1^ activation in non-tumor alveolar parenchyma and in bronchi/bronchioles observed in tamoxifen-treated BRAF^V600E^ mice is an immediate and acute effect of BRAF^V600E^ expression rather than a cumulative effect over time of random events of Cre-dependent recombination of BRAF^V600E^ allele which may lastly result in a basal/chronic BRAF^V600E^-activation.

### Early effects of BRAF^V600E^ expression on leukocytes in lungs

We also checked whether BRAF^V600E^ might elicit acute lung inflammation. Interestingly, 4 days after BRAF^V600E^-induction, we found a dramatic infiltration of neutrophils, harboring the characteristic multilobed nuclei and staining positive for the neutrophilic marker myeloperoxidase (MPO) (Supplementary Fig. 10A, B) in the alveolar parenchyma of BRAF^V600E^ mice but not in controls. Nevertheless, we found no enrichment of cells positive for the monocyte/macrophagic marker F4/80 in the alveoli (Supplementary Fig. 10C). Furthermore, the occurrence of CD4^+^ T-lymphocytes in BRAF^V600E^ mice was reduced twice compared to control (Supplementary Fig. 10D), thus indicating that BRAF^V600E^ ubiquitous expression induces an immediate alveolar infiltration of neutrophils as well as a loss in CD4 + lymphocytes without affecting monocytic/macrophagic lineage.

Conversely, albeit F4/80^+^ cells were increased in BRAF^V600E^ bronchi/bronchioles compared to controls (Supplementary Fig. 10E), neither neutrophils nor CD4^+^lymphocytes frequencies were perturbed (Supplementary Fig. 10F, G), thus suggesting that BRAF^V600E^ triggers an immediate bronchial infiltration specifically of F4/80^+^ cells. Similarly, also adenomas displayed a specific enrichment in F4/80^+^ cells (Supplementary Fig. 10H), but not in either MPO- or CD4-positive cells (Supplementary Fig. 10I, J), thus possibly indicating that BRAF^V600E^-driven adenomas may preferentially recruit macrophages rather than neutrophils or lymphocytes.

**Fig. 7:**
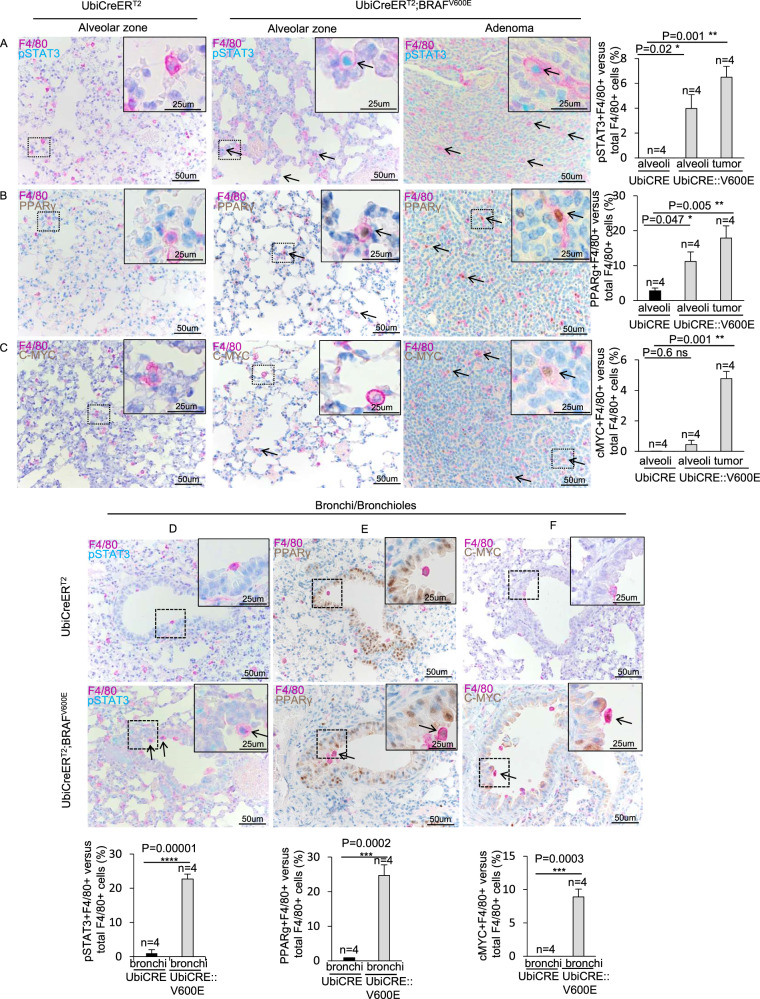
BRAF^V600E^ induction results in an overall M2-like macrophages increase in the lung. **A**–**C** Representative images (on the left) and quantifications (on the right) showing F4/80+ cells staining positive for **A** pSTAT3, **B** PPARγ, **C** c-MYC immunostainings in alveolar parenchyma and adenoma sections from BRAF^V600E^ and control mice. **D**–**F** Representative images (top) and quantifications (bottom) showing F4/80+ cells staining positive for **D** pSTAT3, **E** PPARγ, **F** c-MYC double immunostainings in bronchi/bronchiolar parenchyma of lung sections from BRAF^V600E^ and control mice. Quantifications were performed on at least five different areas of the lung sections in a random way. Data are expressed as mean ± SEM; *n* = animals per group. *P < 0.05; **P < 0.01; ***P < 0.001, ns = not significant. (ANOVA test with Dunnet post-hoc correction (**A**, **B**, **C**), T Student’s test unpaired (**D**, **E**, **F**)). Arrows point to selected positive cells for the indicated marker. Insets: magnifications of areas inside dashed squares.

Furthermore, double immunostainings revealed that the percentages of F4/80^+^ cells positive for the anti-inflammatory M2 macrophagic markers pSTAT3 [33–35] peroxisome-activated proliferator receptor-γ (PPARγ) [33, 36, 37] and c-MYC [33, 38, 39] were globally enhanced in both bronchial/bronchiolar and alveolar parenchyma of mutant mice as well as in adenomas compared to control (Fig. 7A–F), thus indicating a BRAF^V600E^-mediated overall increase in pro-tumoral macrophages in lungs. Conversely, F4/80^+^ cells positive for the pro-inflammatory M1 macrophagic marker hypoxia inducible factor-1α (HIF1α) [35, 40–42] were significantly increased only in adenomas, but not in either alveolar or bronchial/bronchiolar parenchyma of mutant mice compared to controls (Supplementary Fig. 11A, B), thus giving further confirmation on the ability of BRAF^V600E^ to orchestrate the immediate recruitment of specific leukocytes in different pulmonary epithelia.

### BRAF^V600E^-expression induces ROS generation in vivo

**Fig. 8:**
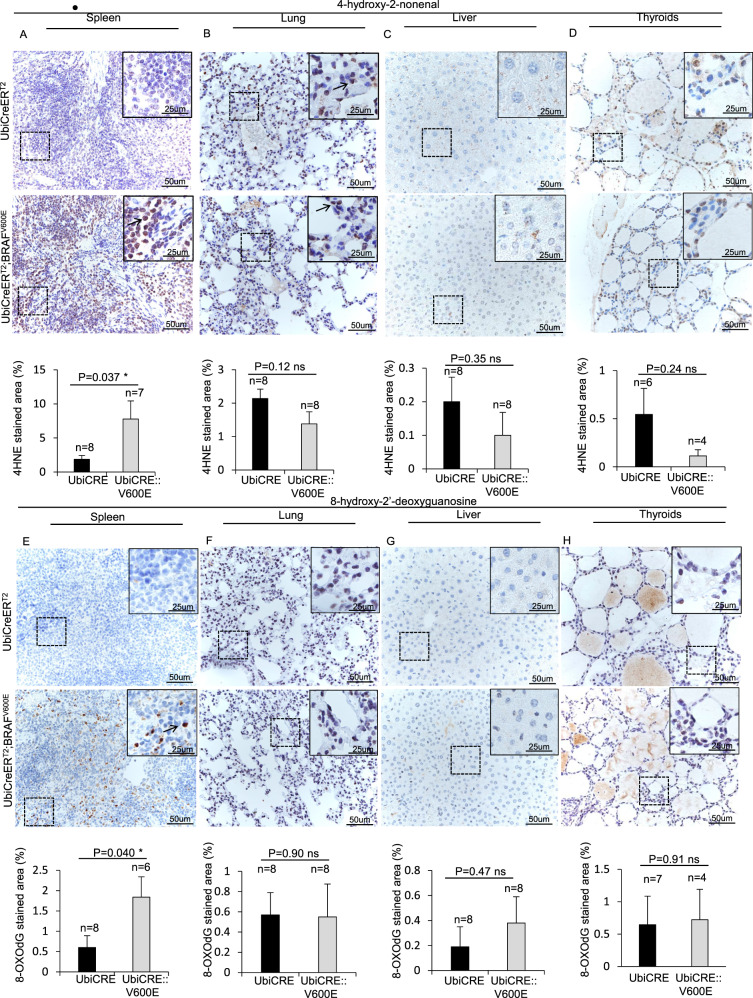
BRAF^V600E^ induction results in ROS generation in the spleen but not in the lungs, liver or thyroids. Representative images (top) and quantifications (bottom) showing stainings positive for **A**–**D** 4-hydroxy-2-nonenal and **E**–**H** 8-hydroxy-2’-deoxyguanosine respectively in spleen (**A, E**), lung (**B, F**), liver (**C, G**) and thyroids (**D, H**) sections from BRAF^V600E^ and control mice. Quantifications were performed on at least five different areas of tissue sections in a random way. Data are expressed as mean ± SEM; *n* = animals per group. *P < 0.05; **P < 0.01; ***P < 0.001, ns = not significant. (T Student’s test unpaired). Arrows point to selected positive cells for the indicated marker. Insets: magnifications of areas inside dashed squares.

We finally checked whether BRAF^V600E^ might result in reactive oxygen species (ROS) production in vivo. Interestingly, immunostaining experiments revealed increased levels of the ROS markers 4-hydroxy-2-nonenal [43–46] and 8-hydroxy-2’-deoxyguanosine [43, 47–50] in the spleen but not in lungs, liver, or thyroids of BRAF^V600E^-mice compared to controls, thus suggesting that, albeit the BRAF^V600E^-induced DNA damage may be ROS-dependent in vivo as well as in vitro [51], additional mechanisms might be involved in BRAF^V600E^-dependent DNA damage induction in lungs and liver (Fig. 8A–H).

## Discussion

Here we reported that ubiquitous acute BRAF^V600E^ expression leads to a rapidly lethal sickness characterized by general weakness and weight loss. This outcome may be partially ascribable to lung acute inflammation and to a rapid energetic depletion likely attributable to BRAF^V600E^-triggered lipolysis, a process which is mediated by activated RAS-pathway [52]. We also observed that BRAF^V600E^ activation induces microvesicular HS, a condition where an excess of fatty acids is accumulated into hepatocytes. Thus, it is conceivable that the BRAF^V600E^-triggered hepatic fat accumulation may be ascribable to a likely increase of serum fatty acids consequent to the RAS-pathway-driven lipolysis in adipocytes [52].

Our findings provided the first evidence in vivo that acute BRAF^V600E^ expression elicits instant DNA damage in an organ-specific fashion. p21^CIP1^ [53], which may be activated by p53 upon genotoxic insults [54] and by oncogene activation *via* pRb/E2F [22], promotes cell cycle arrest and senescence [22] by inhibiting CDKs [22]. Nevertheless, despite BRAF^V600E^ induces both DNA damage and p21^CIP1^ activation in vitro [51, 55] as well as in senescent lung adenomas [17], we found no differences in p21^CIP1^ levels either in liver or spleen upon BRAF^V600E^ expression. Thus, we unveiled that, in the organs where BRAF^V600E^ rapidly induces robust DNA damage, an immediate p21^CIP1^ activation does not occur in a generalized manner. Such observations suggest that p21^CIP1^ may be activated only at later time points in the presence of a constant oncogenic stimulus, or that BRAF^V600E^ ability to induce DNA damage in certain tissues/organs may be uncoupled from p21^CIP1^ activation.

We also uncovered that BRAF^V600E^ expression yields a differential response of cell cycle/senescence-associated proteins in ATIIs. Indeed, albeit all the BRAF^V600E^-challenged ATIIs showed increased DNA damage, while non-transformed ATIIs express p21^CIP1^ in the absence of p53 activation, tumorigenic ATIIs displayed enhanced p53 expression coupled with a significant p21^CIP1^reduction compared to non-tumor cells. The strike differences in such expression patterns argue for the possibility that non-tumor ATIIs may represent an early stage of tumor development in which a rapid p53-independent-p21^CIP1^ induction might be an immediate barrier to cancer initiation which, at a certain point, may be repressed thus allowing cell proliferation. Alternatively, some ATIIs might be naturally more refractory to an immediate BRAF^V600E^-dependent p21^CIP1^ activation and be more prone to give rise to adenomas, which lastly, during the onset of senescence, will activate p53. Thus, the evidence that p53 induction in adenoma ATIIs is accompanied by no alteration in p21 expression might be due to the fact the p53 activation is at an initial stage and therefore it has not reached yet the threshold necessary for an efficient p21^CIP1^ gene activation.

Albeit it has been established that BRAF^V600E^ promotes senescence or apoptosis without yielding any previous proliferative stimulation in vitro [51, 56], BRAF^V600E^ expression in vivo results in an initial hyperplasic wave lastly culminating in the onset of tumor senescence, characterized by DNA damage [17], and p21^CIP1^ [17], p19^ARF^ [9], p16^INK4a^ [21] and p53 [57] expression. Nevertheless, we unveiled that in BRAF^V600E^ mice, non-tumor alveolar parenchyma showed rapid p21^CIP1^ induction, which is not accompanied by activation of any among the well-known proteins associated to OIS in lungs [9, 17], thus arguing for the possibility that such immediate tumor-suppression response may differ from classical OIS. Indeed, such p53/pRb-independent p21^CIP1^ activation in non-tumor alveolar parenchyma may reflect a rapid BRAF^V600E^-mediated cytotoxic response reminiscent to that observed in CCs (see below), thus suggesting that albeit BRAF^V600E^ ATIIs are prone to give rise to adenomas, yet there is an immediate cell cycle arrest in a minority of the challenged ATIIs.

In contrast to the extensive research work conducted in ATIIs, very little is known about the early molecular effects of BRAF^V600E^ in bronchi/bronchioles. Here we unveiled that BRAF^V600E^ initiates CCs transdifferentiation into ATIIs. Concomitantly, we observed a proliferation stimulation resulting in DNA damage, cell cycle arrest and cell death. Both proliferative and cytotoxic responses are much more exacerbated in CCs compared to ATIIs, which can account for the well-known characteristic of CCs to be recalcitrant to RAS-pathway stimulation [58, 59].

We also uncovered that BRAF^V600E^ rapidly elicits an acute inflammatory response in lungs by differentially recruiting neutrophils in the alveoli and F4/80-positive cells in bronchi/bronchioles and adenomas. The generation of GEMMs in which BRAF^V600E^ expression is driven specifically in neutrophils and alveolar macrophages will provide helpful insights into the mechanisms underlying the BRAF^V600E^ pleiotropic effect on leukocytes in lungs.

## Materials and methods

### Murine models

BRAF^LSLV600E^ mice were described previously [9, 10, 12]. This mouse model was crossed with a mouse strain carrying ubiquitously expressed, tamoxifen-activated recombinase, UBC-CreER^T2^ [18], to generate UBC-CreER^T2/+^;BRAF^LSL_V600E/+^ mice. These mice received intraperitoneal injections of 4-hydroxy tamoxifen (Sigma H6278) (1 mg/injection, 3–4 injections, 1 injection per day for 3 or 4 consecutive days).

All mice were maintained at the Spanish National Cancer Research Centre under specific pathogen-free conditions in accordance with the recommendations of the Federation of European Laboratory Animal Science Associations (FELASA). All animal experiments were approved by our Institutional Animal Care and Use Committee (IACUC) and by the Ethical Committee for animal experimentation (CEIyBA) (PROEX 106.7/20). We followed the Reporting in Vivo Experiments (ARRIVE) guidelines developed by the National Centre for the Replacement, Refinement & Reduction of Animals in Research (NC3Rs). Both male and female mice, with mixed background, were used for the experiments.

### Immuno-FISH

Immuno-FISH was performed in formalin-fixed paraffin-embedded mouse lung sections to identify telomeric induced foci (TIF) as previously described [60, 61]. Immuno-FISH was performed as follows: after deparaffination and citrate antigen retrieval, samples were permeabilized for 3 h in PBS1X-0.5% Triton, blocked for 2 h with 10% fetal bovine serum and 1 h with 5% BSA in PBS1X-0.1%Triton-10mM Glycine (PBSTG), and immunofluorescence with anti-53BP1 rabbit antibody (Novus Biologicals NB100-304) diluted 1:500 was performed. Samples were incubated O/N at 4 °C with the primary antibody in PBSTG. Slides were further washed with PBSTG and incubated with 488-Alexa labeled secondary antibody in DAKO antibody diluent reagent (S3022). After immunofluorescence, samples were fixed for 20 min in 4% paraformaldehyde in PBS1X and followed by FISH. Briefly, samples were washed with PBS and dehydrated in Ethanol 70, 90 and 100%. The samples were then incubated with a telomeric PNA probe labeled with CY3 (Panagene) in 50% formamide for 30 min, washed in the presence of 50% formamide and counterstained with DAPI. TIF were identified by colocalization of CY3 and 488-Alexa double positive spots. Confocal microscopy was performed at room temperature with a laser-scanning microscope (TCS SP5; Leica) using a Plan Apo 63Å-1.40 NA oil immersion objective (HCX; Leica). Maximal projection of Z-stack images generated using advanced fluorescence software (LAS) was analyzed with the Definiens XD software package. The DAPI images were used to detect signals inside the nuclei.

### Immunohistochemistry analyses in tissue sections

Tissues were fixed in 10% buffered formalin, embedded in paraffin wax and sectioned at 5 mm. For histological examination sections were stained with hematoxylin and eosin, according to standard procedures as previously described [62]. CC3 Cleaved Caspase 3 Asp175 (Cell Signaling Technology 9661), CC10 (Santa Cruz Biotechnology sc-9772), CD4 (Cell Signaling Technology 25229, prosurfactant protein C (millipore AB3786), p21 (291 H/B5, homemade), γH2AX Ser 139 (Millipore 05-636), PPERK Thr202/Tyr204 (Cell Signaling Tehcnology 9378), Ki67 (Cell Signaling 12202), MPO (Dako A0398), F4-80 (ABD Serotec MCA497), p16 (33B, homemade), p19 ARF (sc-32748 Santa Cruz), pRb (ser807/811, #9308, Cell Signaling), pSMAD3 (ser423/425 ab52903, Abcam), pSTAT3 (tyr705, #9145 Cell Signaling), p53 (POE316A, homemade), cyclin D1 (M3635, Dako), c-MYC (ab32072, Abcam), PPARγ (Cell Signaling, #2435), HIF1α (Cell Signaling, #36169), 8-hydroxy-2’-deoxyguanosine (Abcam, ab48508), 4-hydroxy-2-nonenal (Alpha Diagnostic, HNE11-S) antibodies were used for immunohistochemistry in tissue sections. Pictures were taken using Olympus AX70 microscope. The percentage of positive cells was identified by eye and the areas were calculated by ImageJ and Zen 3.1 (Zeiss) softwares.

### Protein extract preparation and Western blot

Protein extracts were obtained as follows: 45 mg of lung for each mouse were mechanically homogenized in 850ul lysis buffer (50 mM TrisHCl pH 7.5, 420 mM NaCl, 1% Triton, 1 mM EDTA, 2.5 mM MgCl2, protease inhibitors) in *BERTIN Precellys 24 Lysis & Homogenization* machine, incubated 30 min on ice in agitation, sonicated 10 sec, centrifuged at 14000 g for 20 min at 4 °C. The recovered supernatant was passed through a 0.22 filter, aliquoted, flash-frozen in liquid nitrogen and stored at −80 °C. Protein concentration was determined using the Bio‐Rad DC Protein Assay (Bio‐Rad). 40 µg of nuclear protein extracts were separated in SDS–polyacrylamide gels by electrophoresis. After protein transfer onto nitrocellulose membrane, the membranes were incubated with the indicated antibodies: monoclonal anti-actin 1:5000 (A5441, Sigma), anti-BRAF 1:200 (F-7, sc-5284, Santa Cruz), anti-BRAF^V600E^ 1:300 (31-1042-00 RevMAB Biosciences USA), anti-γH2AX Ser139 1:5000 (Merck 05-636), homemade rat anti-p15^INK4b^ clone PAT65B (neat supernatant), homemade rat anti-p16^INK4a^ clone PABLO33B (neat supernatant), handmade rat anti-p19^ARF^ clone PIL346C (neat supernatant), homemade rat anti-p27^KIP1^ clone SON82D (neat supernatant), homemade rat anti-p21^CIP1^ clone HUGO291 (neat supernatant), homemade rat anti-p53 clone POE316A (neat supernatant). Antibody binding was detected after incubation with a secondary antibody coupled to horseradish peroxidase using chemiluminescence with ECL detection KIT (GE Healthcare) with Chemidoc (Biorad). For the quantification, protein‐band intensities were quantified by densitometric analysis with ImageLab software (Biorad). The total levels of each protein analyzed have been normalized versus actin and the mean of the specific protein/actin ratio deriving from at least 3 different replicates has been used to generate the chart as previously described [63].

### PCR

DNA of tissue samples was extracted using Phenol:Chloroform:Isoamyl:Alcohol (Sigma). We determined Cre-mediated recombination by using the following PCR program: 94 °C for 3 min, followed by 33 cycles of 94 °C denaturation for 25 s, 25 s annealing at 55 °C, elongation at 73 °C for 45 s, followed by a 4 min 73 °C elongation step with the following primes: Fw 5’-TGAGTATTTTTGTGGCAACTGC and Rev 5’-CTCTGCTGGGAAAGCGGC. This oligonucleotide primer pair hybridizes in intron 14 flanking the cassette insertion site. These conditions produce diagnostic PCR products of 185 bp for the wild-type BRAF and 308 bp for BRAF^V600E^alleles and a 335 bp PCR product for the Cre-activated BRAF^V600E^ allele. The samples were resolved in a 3% agarose gel.

### Quantification and statistical analysis

Immunohistochemistry quantifications were performed by direct cell counting by using Zen3.1 Zeiss and Image J softwares. ImmunoFISH quantifications were carried out by direct counting of cells and 53BP1 foci on single plans of each z-stack by using LAS X software (Leica). Unpaired Student’s *t*-test (two-tailed), ANOVA followed by Tukey’s post-hoc correction, Log Rank test were used to determine statistical significance. *P*-values of less than 0.05 were considered significant. *p < 0.05, **p < 0.01, ***p < 0.001. Statistical analysis was performed using Microsoft® Excel 2016 and GraphPad/PRISM8. For animal studies no blinding/randomization was done/used. The number of mice per each experiment as well as the size of the experiments were obtained by performing power analysis.

## Supplementary information

Supplementary Figure 1

Supplementary Figure 2

Supplementary Figure 3

Supplementary Figure 4

Supplementary Figure 5

Supplementary Figure 6

Supplementary Figure 7

Supplementary Figure 8

Supplementary Figure 9

Supplementary Figure 10

Supplementary Figure 11

Supplementary Figure Legends

Materials and Methods

Agreements and responses from co-authors with change

Reproducibility Checklist

## Data Availability

The datasets and other information that support the findings of this study are available from the corresponding author upon reasonable request.
