# Supplementary material for: Early differential responses elicited by BRAFV600E in adult mouse models
Source: Cell Death Dis. 2022 Feb 10;13(2):142. doi: 10.1038/s41419-022-04597-z (PMC8831492; doi:10.1038/s41419-022-04597-z)
Supplement: Supplementary file 1 — Supplementary Figure 1 [file 41419_2022_4597_MOESM1_ESM.pptx]

## Slide 1
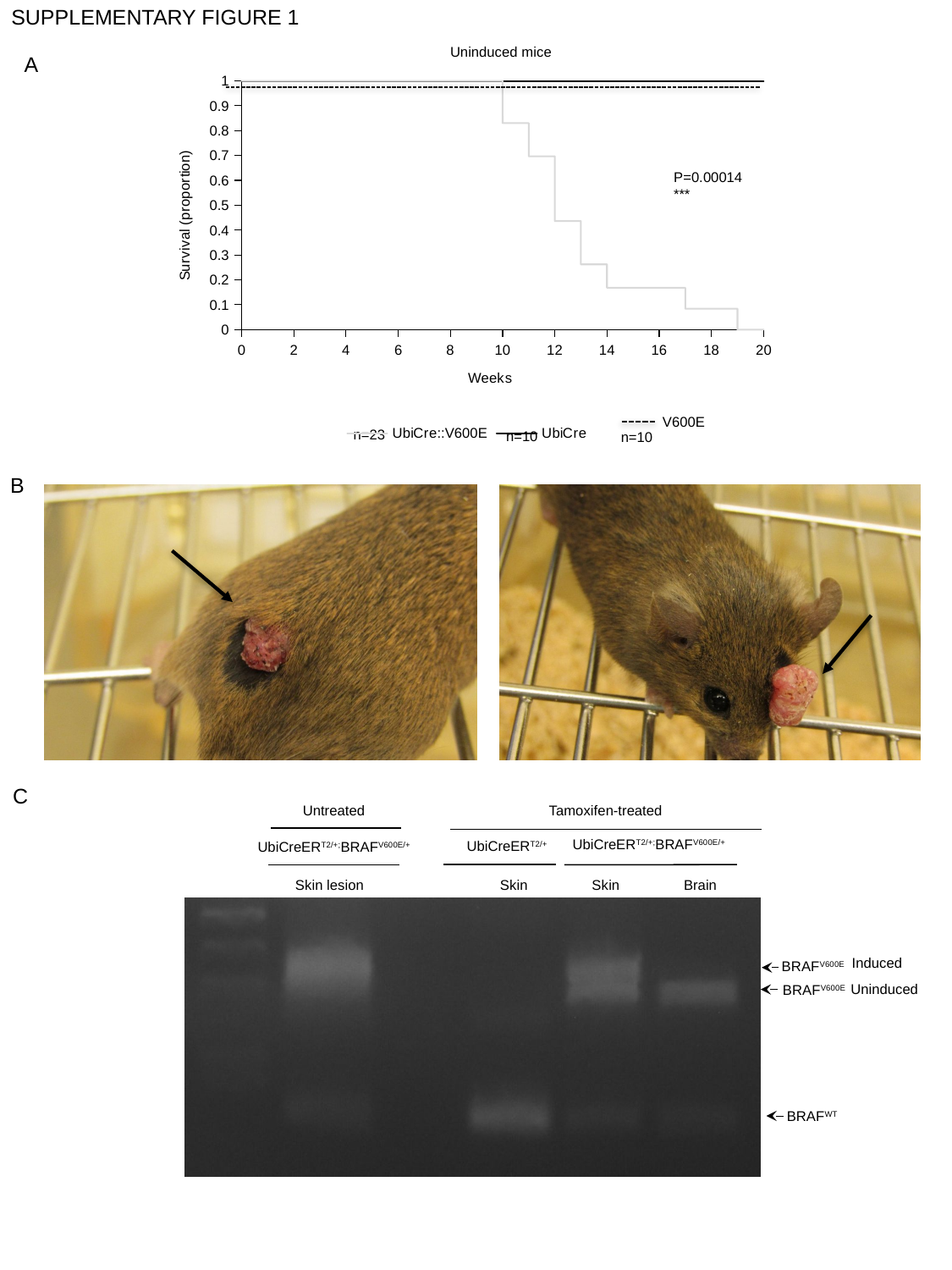

SUPPLEMENTARY FIGURE 1
Uninduced mice
A
### Chart
| Category | UbiCre::V600E | UbiCre |
|---|---|---|P=0.00014
***
V600E
n=23
n=10
n=10
B
C
Untreated Tamoxifen-treated
 UbiCreERT2/+;BRAFV600E/+
 UbiCreERT2/+
 UbiCreERT2/+;BRAFV600E/+
 Skin lesion Skin Skin Brain
Induced
BRAFV600E
Uninduced
BRAFV600E
BRAFWT
