# Supplementary material for: Early differential responses elicited by BRAFV600E in adult mouse models
Source: Cell Death Dis. 2022 Feb 10;13(2):142. doi: 10.1038/s41419-022-04597-z (PMC8831492; doi:10.1038/s41419-022-04597-z)
Supplement: Supplementary file 2 — Supplementary Figure 2 [file 41419_2022_4597_MOESM2_ESM.pptx]

## Slide 1
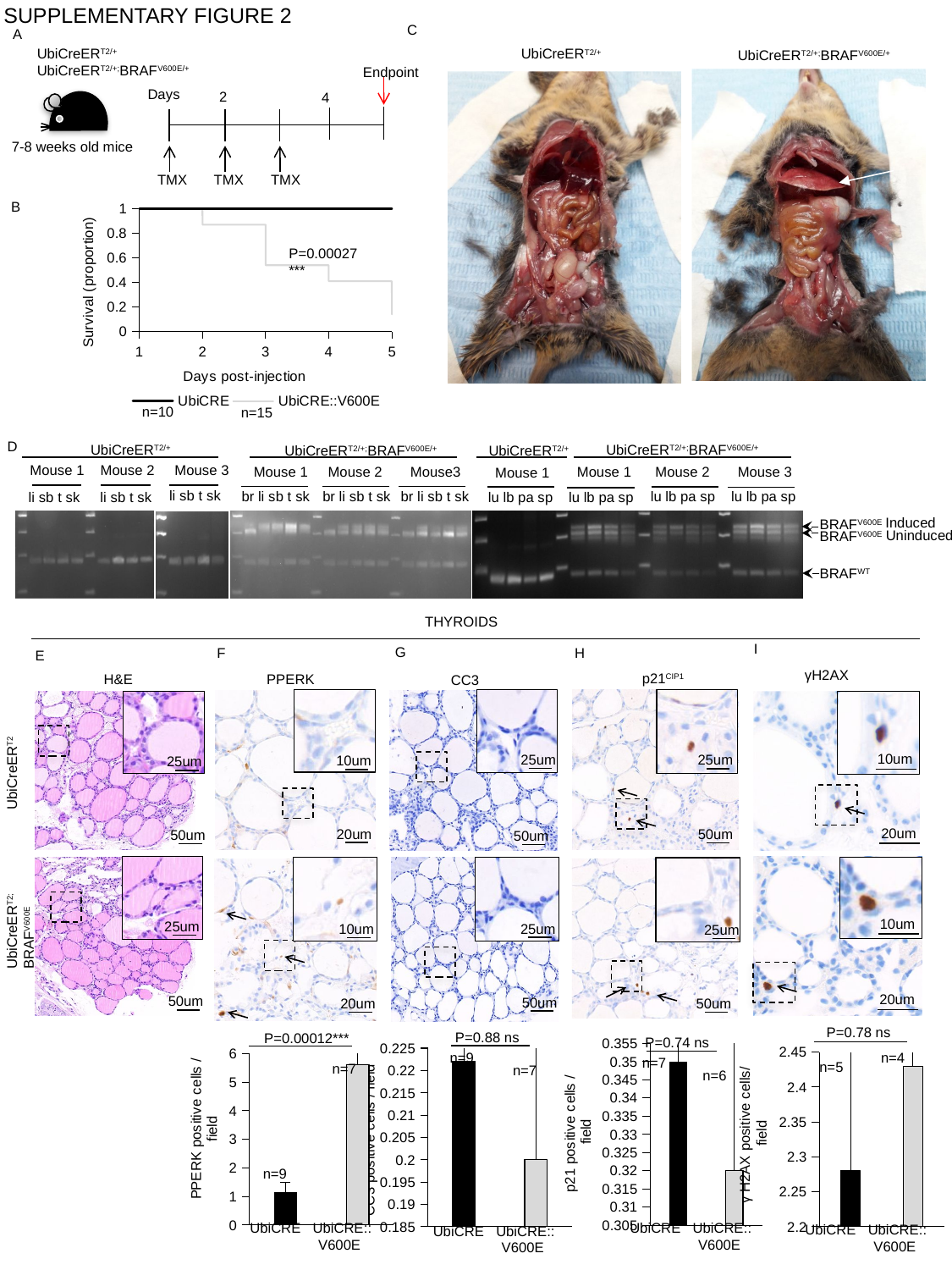

SUPPLEMENTARY FIGURE 2
UbiCreERT2/+
UbiCreERT2/+;BRAFV600E/+
C
A
UbiCreERT2/+
 UbiCreERT2/+;BRAFV600E/+
Endpoint
Days
2
4
7-8 weeks old mice
TMX
TMX
TMX
B
### Chart
| Category | UbiCRE | UbiCRE::V600E |
|---|---|---|P=0.00027
***
n=10
n=15
D
 UbiCreERT2/+;BRAFV600E/+
 UbiCreERT2/+
 UbiCreERT2/+
 UbiCreERT2/+;BRAFV600E/+
Mouse 1 Mouse 2 Mouse 3
Mouse 1 Mouse 2 Mouse 3
Mouse 1 Mouse 2 Mouse3
Mouse 1
li sb t sk
br li sb t sk
 lu lb pa sp lu lb pa sp
br li sb t sk
br li sb t sk
li sb t sk li sb t sk
 lu lb pa sp lu lb pa sp
Induced
BRAFV600E
Uninduced
BRAFV600E
BRAFWT
THYROIDS
I
G
H
F
E
γH2AX
p21CIP1
H&E
PPERK
CC3
UbiCreERT2
10um
25um
25um
10um
25um
20um
20um
50um
50um
50um
UbiCreERT2;
BRAFV600E
10um
25um
25um
10um
25um
20um
50um
50um
50um
20um
P=0.78 ns
P=0.88 ns
P=0.00012***
P=0.74 ns
### Chart
| Category | |
|---|---|
| UbiCRE | 0.35 |
| UbiCRE::V600E | 0.32 |
### Chart
| Category | |
|---|---|
| UbiCRE | 0.222 |
| UbiCRE::V600E | 0.2 |
### Chart
| Category | |
|---|---|
| UbiCRE | 2.28 |
| UbiCRE::V600E | 2.43 |
### Chart
| Category | |
|---|---|
| UbiCRE | 1.16 |
| UbiCRE::V600E | 5.6 |n=9
n=4
n=7
n=5
n=7
n=7
n=6
γ H2AX positive cells/
field
n=9
UbiCRE UbiCRE::
 V600E
UbiCRE UbiCRE::
 V600E
UbiCRE UbiCRE::
 V600E
UbiCRE UbiCRE::
 V600E
