# Supplementary material for: Early differential responses elicited by BRAFV600E in adult mouse models
Source: Cell Death Dis. 2022 Feb 10;13(2):142. doi: 10.1038/s41419-022-04597-z (PMC8831492; doi:10.1038/s41419-022-04597-z)
Supplement: Supplementary file 3 — Supplementary Figure 3 [file 41419_2022_4597_MOESM3_ESM.pptx]

## Slide 1
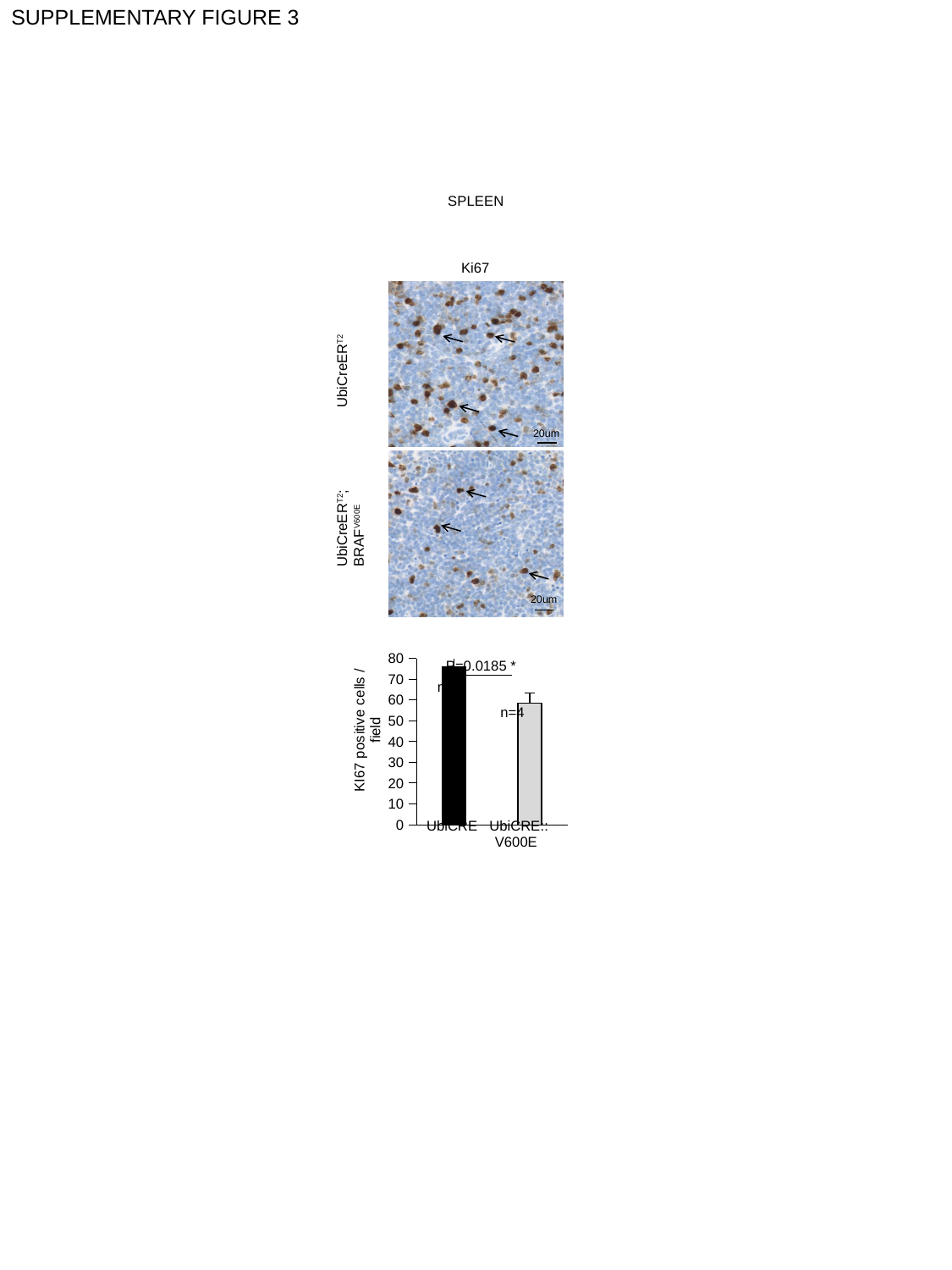

SUPPLEMENTARY FIGURE 3
SPLEEN
Ki67
UbiCreERT2
20um
UbiCreERT2;
BRAFV600E
20um
### Chart
| Category | |
|---|---|
| UbiCRE | 75.8 |
| UbiCRE::V600E | 58.5 |P=0.0185 *
n=5
n=4
UbiCRE UbiCRE::
 V600E
