# Supplementary material for: Early differential responses elicited by BRAFV600E in adult mouse models
Source: Cell Death Dis. 2022 Feb 10;13(2):142. doi: 10.1038/s41419-022-04597-z (PMC8831492; doi:10.1038/s41419-022-04597-z)
Supplement: Supplementary file 4 — Supplementary Figure 4 [file 41419_2022_4597_MOESM4_ESM.pptx]

## Slide 1
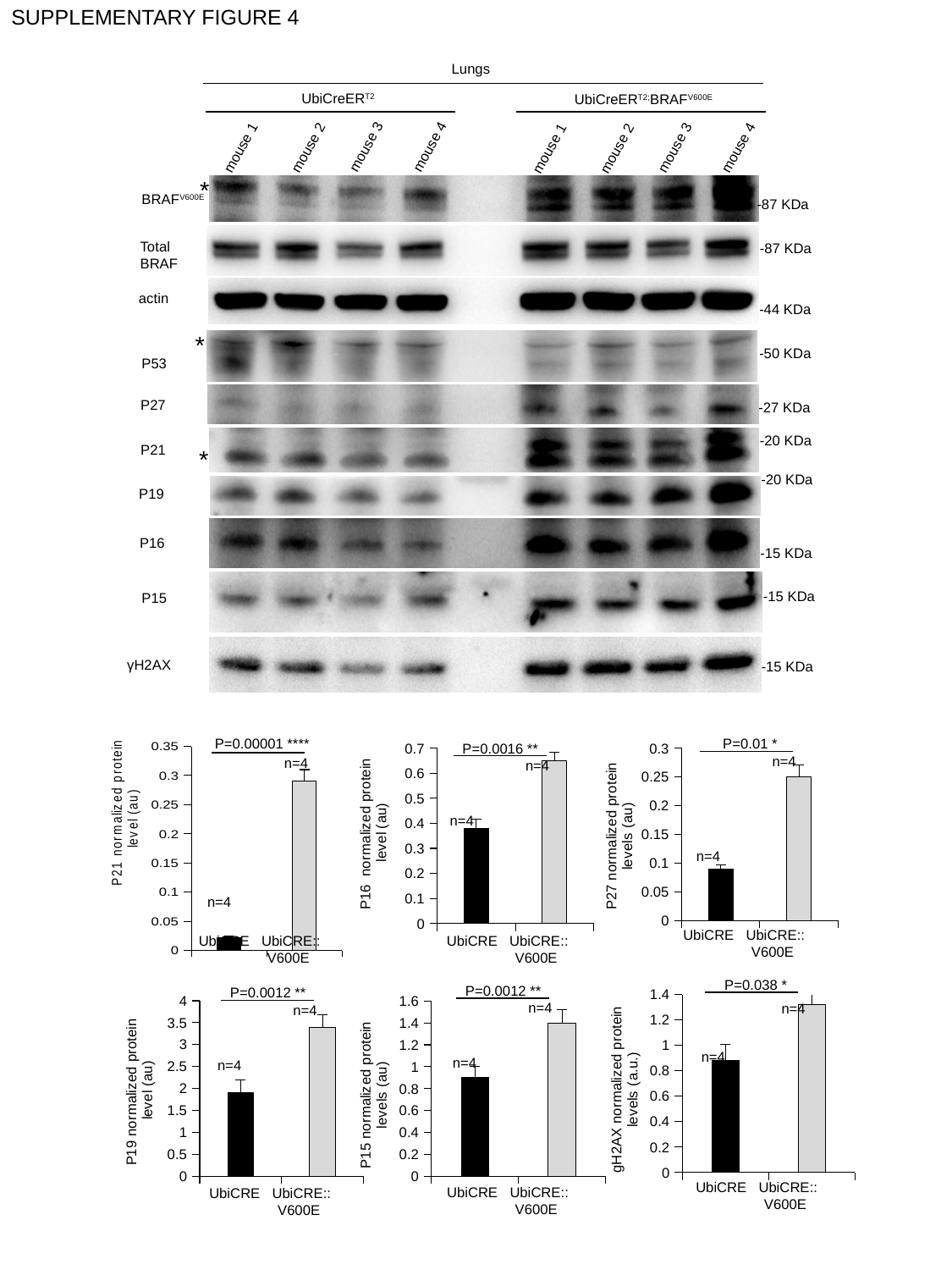

SUPPLEMENTARY FIGURE 4
Lungs
UbiCreERT2
UbiCreERT2;BRAFV600E
mouse 3
mouse 4
mouse 3
mouse 1
mouse 2
mouse 4
mouse 1
mouse 2
*
BRAFV600E
-87 KDa
Total
BRAF
-87 KDa
actin
-44 KDa
*
-50 KDa
P53
P27
-27 KDa
-20 KDa
P21
*
-20 KDa
P19
P16
-15 KDa
-15 KDa
P15
γH2AX
-15 KDa
P=0.00001 ****
P=0.01 *
P=0.0016 **
### Chart
| Category | |
|---|---|
| UbiCRE | 0.022 |
| UbiCRE::V600E | 0.29 |
### Chart
| Category | |
|---|---|
| UbiCRE | 0.38 |
| UbiCRE::V600E | 0.65 |
### Chart
| Category | |
|---|---|
| UbiCRE | 0.09 |
| UbiCRE::V600E | 0.25 |n=4
n=4
n=4
n=4
n=4
n=4
UbiCRE UbiCRE::
 V600E
UbiCRE UbiCRE::
 V600E
UbiCRE UbiCRE::
 V600E
P=0.038 *
P=0.0012 **
P=0.0012 **
### Chart
| Category | |
|---|---|
| UbiCRE | 0.88 |
| UbiCRE::V600E | 1.32 |
### Chart
| Category | |
|---|---|
| UbiCRE | 1.9 |
| UbiCRE::V600E | 3.4 |
### Chart
| Category | |
|---|---|
| UbiCRE | 0.9 |
| UbiCRE::V600E | 1.4 |n=4
n=4
n=4
n=4
n=4
n=4
UbiCRE UbiCRE::
 V600E
UbiCRE UbiCRE::
 V600E
UbiCRE UbiCRE::
 V600E
