# Supplementary material for: Early differential responses elicited by BRAFV600E in adult mouse models
Source: Cell Death Dis. 2022 Feb 10;13(2):142. doi: 10.1038/s41419-022-04597-z (PMC8831492; doi:10.1038/s41419-022-04597-z)
Supplement: Supplementary file 5 — Supplementary Figure 5 [file 41419_2022_4597_MOESM5_ESM.pptx]

## Slide 1
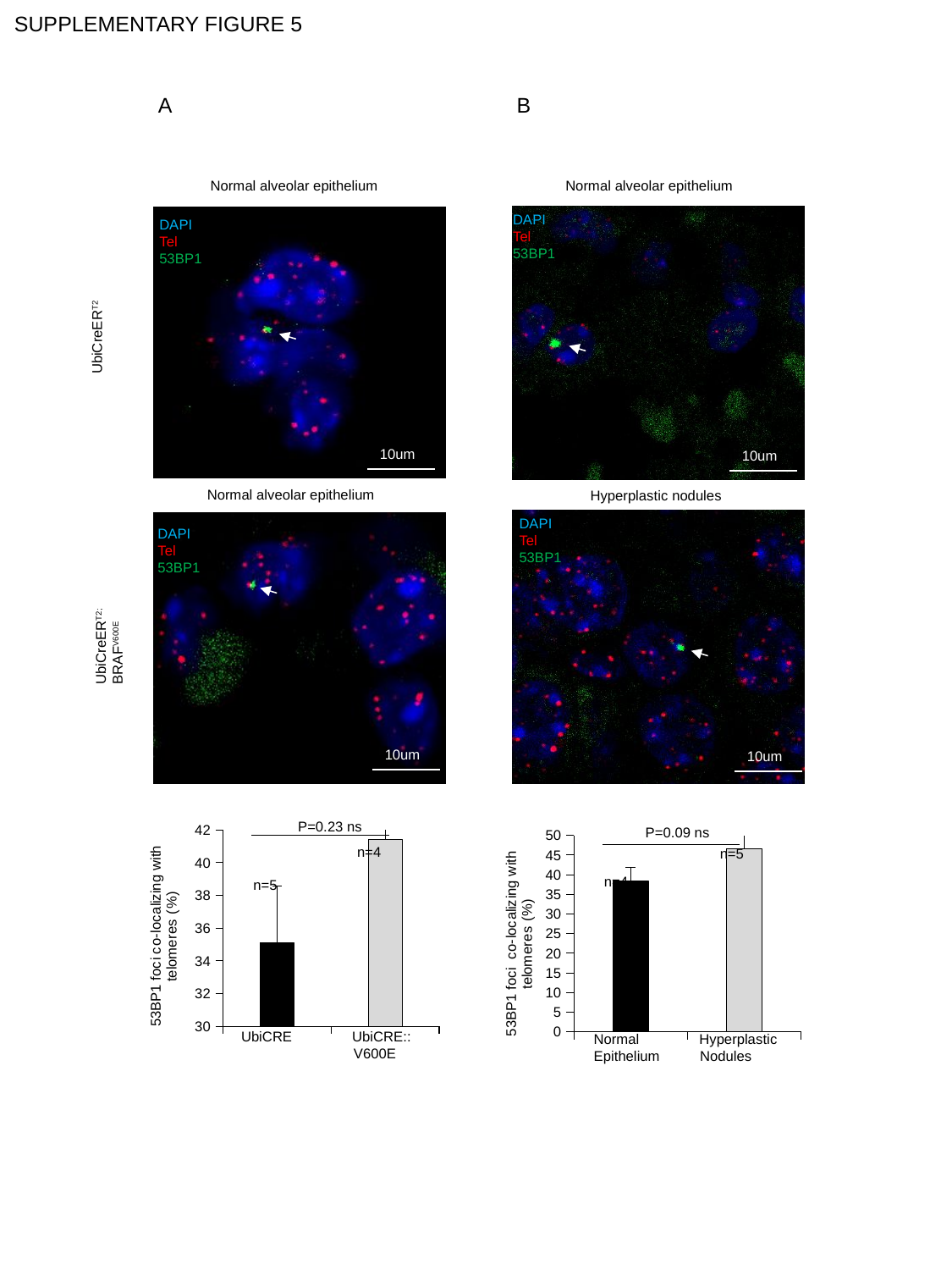

SUPPLEMENTARY FIGURE 5
B
A
Normal alveolar epithelium
Normal alveolar epithelium
DAPI
Tel
53BP1
DAPI
Tel
53BP1
UbiCreERT2
10um
10um
Normal alveolar epithelium
Hyperplastic nodules
DAPI
Tel
53BP1
DAPI
Tel
53BP1
UbiCreERT2;
BRAFV600E
10um
10um
P=0.23 ns
### Chart
| Category | |
|---|---|
| UbiCRE | 35.08 |
| UbiCRE:V600E | 41.4 |P=0.09 ns
### Chart
| Category | |
|---|---|
| Normal tissue | 38.3 |
| Lung Tumors | 46.65 |n=4
n=5
n=4
n=5
UbiCRE UbiCRE::
 V600E
 Normal Hyperplastic
 Epithelium Nodules
