# Supplementary material for: Early differential responses elicited by BRAFV600E in adult mouse models
Source: Cell Death Dis. 2022 Feb 10;13(2):142. doi: 10.1038/s41419-022-04597-z (PMC8831492; doi:10.1038/s41419-022-04597-z)
Supplement: Supplementary file 6 — Supplementary Figure 6 [file 41419_2022_4597_MOESM6_ESM.pptx]

## Slide 1
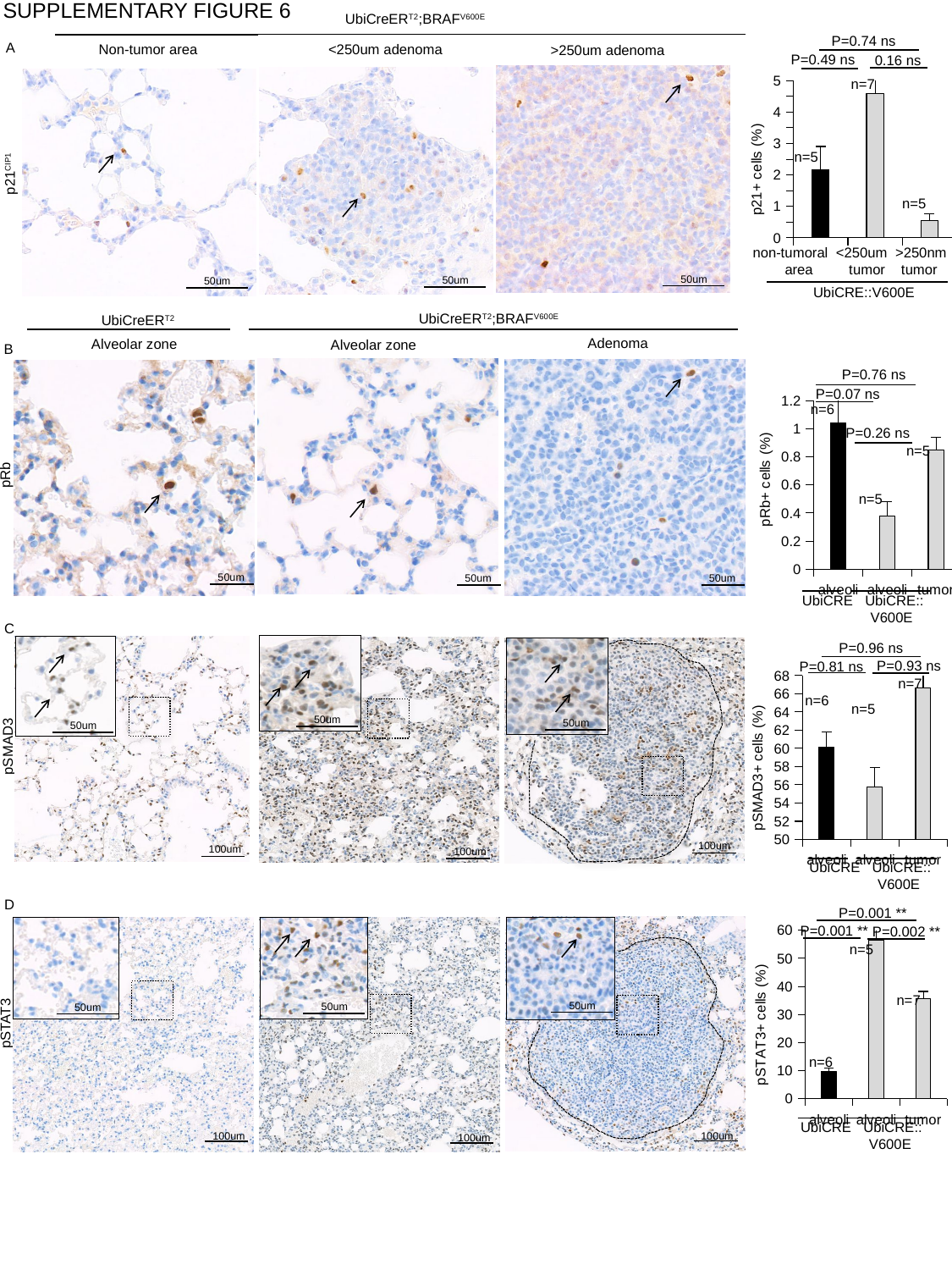

SUPPLEMENTARY FIGURE 6
UbiCreERT2;BRAFV600E
P=0.74 ns
A
Non-tumor area
<250um adenoma
>250um adenoma
P=0.49 ns
0.16 ns
### Chart
| Category | |
|---|---|
| non tumoral area | 2.17 |
| <250um tumor | 4.59 |
| >250um tumor | 0.55 |
n=7
n=5
p21CIP1
n=5
non-tumoral <250um >250nm
 area tumor tumor
50um
50um
50um
UbiCRE::V600E
UbiCreERT2;BRAFV600E
UbiCreERT2
Adenoma
Alveolar zone
Alveolar zone
B
P=0.76 ns
P=0.07 ns
### Chart
| Category | |
|---|---|
| alveoli | 1.04 |
| alveoli | 0.38 |
| tumor | 0.85 |n=6
P=0.26 ns
n=5
pRb
n=5
50um
50um
50um
 UbiCRE UbiCRE::
 V600E
C
P=0.96 ns
P=0.93 ns
P=0.81 ns
### Chart
| Category | |
|---|---|
| alveoli | 60.125 |
| alveoli | 55.8 |
| tumor | 66.6 |n=7
n=6
n=5
50um
50um
50um
pSMAD3
100um
100um
100um
 UbiCRE UbiCRE::
 V600E
D
P=0.001 **
P=0.001 **
P=0.002 **
### Chart
| Category | |
|---|---|
| alveoli | 9.720833333333333 |
| alveoli | 56.395 |
| tumor | 35.6 |n=5
n=7
50um
50um
50um
50um
pSTAT3
n=6
 UbiCRE UbiCRE::
 V600E
100um
100um
100um
