# Supplementary material for: Early differential responses elicited by BRAFV600E in adult mouse models
Source: Cell Death Dis. 2022 Feb 10;13(2):142. doi: 10.1038/s41419-022-04597-z (PMC8831492; doi:10.1038/s41419-022-04597-z)
Supplement: Supplementary file 7 — Supplementary Figure 7 [file 41419_2022_4597_MOESM7_ESM.pptx]

## Slide 1
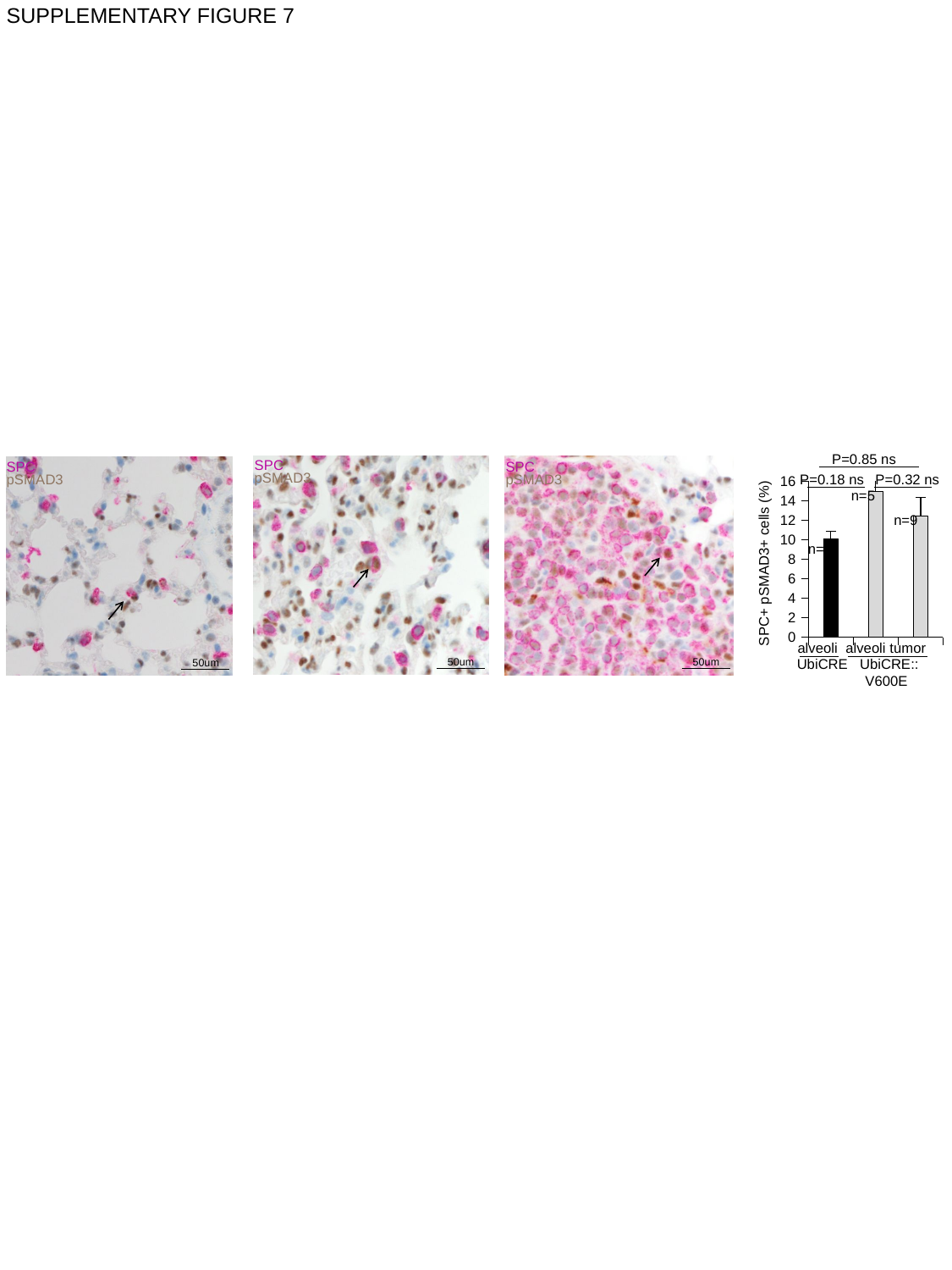

SUPPLEMENTARY FIGURE 7
P=0.85 ns
SPC
SPC
SPC
pSMAD3
pSMAD3
pSMAD3
P=0.18 ns
P=0.32 ns
### Chart
| Category | |
|---|---|
| alveoli | 10.090833333333334 |
| alveoli | 14.913 |
| tumor | 12.441666666666666 |n=5
n=9
n=6
SPC+ pSMAD3+ cells (%)
alveoli alveoli tumor
50um
50um
 UbiCRE UbiCRE::
 V600E
50um
