# Supplementary material for: Early differential responses elicited by BRAFV600E in adult mouse models
Source: Cell Death Dis. 2022 Feb 10;13(2):142. doi: 10.1038/s41419-022-04597-z (PMC8831492; doi:10.1038/s41419-022-04597-z)
Supplement: Supplementary file 8 — Supplementary Figure 8 [file 41419_2022_4597_MOESM8_ESM.pptx]

## Slide 1
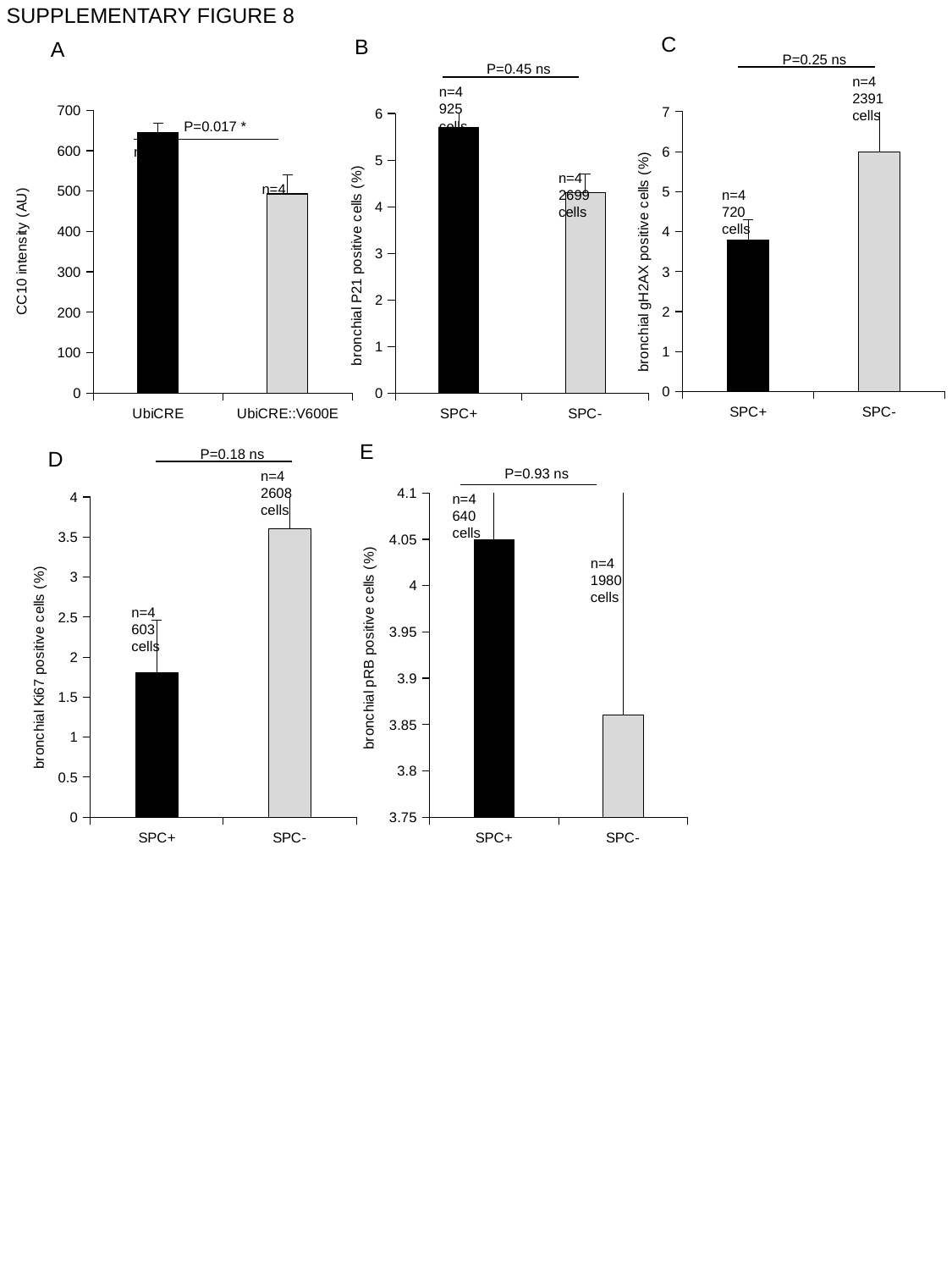

SUPPLEMENTARY FIGURE 8
C
B
A
P=0.25 ns
P=0.45 ns
n=4
2391 cells
n=4
925 cells
### Chart
| Category | |
|---|---|
| UbiCRE | 646.0 |
| UbiCRE::V600E | 493.0 |
### Chart
| Category | |
|---|---|
| SPC+ | 3.8 |
| SPC- | 6.0 |
### Chart
| Category | |
|---|---|
| SPC+ | 5.7 |
| SPC- | 4.3 |P=0.017 *
n=5
n=4
2699 cells
n=4
n=4
720 cells
E
D
P=0.18 ns
P=0.93 ns
n=4
2608 cells
### Chart
| Category | |
|---|---|
| SPC+ | 4.05 |
| SPC- | 3.86 |
### Chart
| Category | |
|---|---|
| SPC+ | 1.8 |
| SPC- | 3.6 |n=4
640 cells
n=4
1980 cells
n=4
603 cells
