# Supplementary material for: Early differential responses elicited by BRAFV600E in adult mouse models
Source: Cell Death Dis. 2022 Feb 10;13(2):142. doi: 10.1038/s41419-022-04597-z (PMC8831492; doi:10.1038/s41419-022-04597-z)
Supplement: Supplementary file 9 — Supplementary Figure 9 [file 41419_2022_4597_MOESM9_ESM.pptx]

## Slide 1
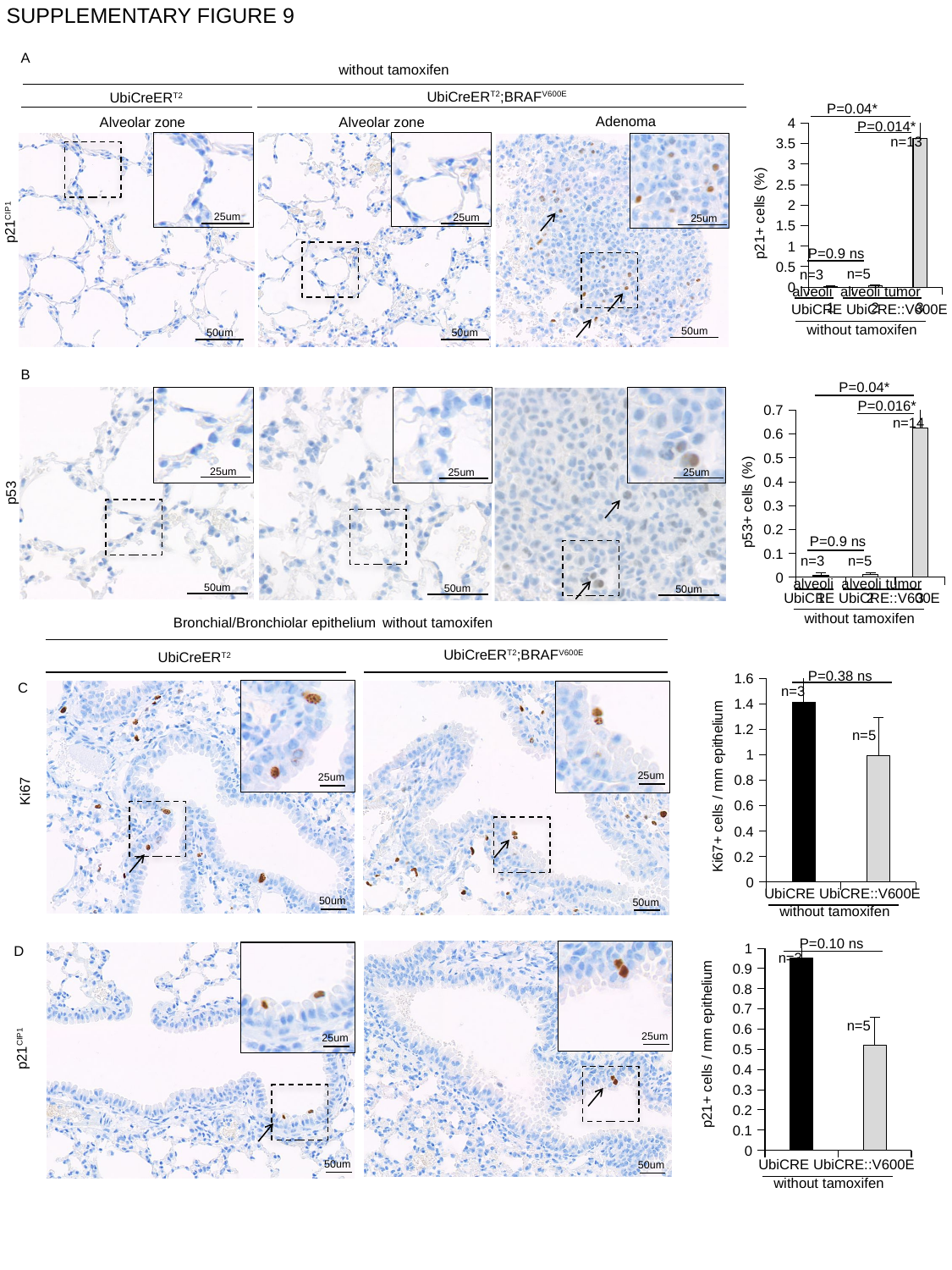

SUPPLEMENTARY FIGURE 9
A
without tamoxifen
UbiCreERT2;BRAFV600E
UbiCreERT2
P=0.04*
Adenoma
Alveolar zone
Alveolar zone
P=0.014*
### Chart
| Category | |
|---|---|n=13
25um
25um
25um
p21CIP1
P=0.9 ns
n=5
n=3
alveoli alveoli tumor
 UbiCRE UbiCRE::V600E
without tamoxifen
50um
50um
50um
B
P=0.04*
P=0.016*
### Chart
| Category | |
|---|---|n=14
25um
25um
25um
p53
P=0.9 ns
n=5
n=3
alveoli alveoli tumor
50um
50um
50um
 UbiCRE UbiCRE::V600E
without tamoxifen
without tamoxifen
Bronchial/Bronchiolar epithelium
UbiCreERT2;BRAFV600E
UbiCreERT2
P=0.38 ns
### Chart
| Category | |
|---|---|
| UbiCRE | 1.4173333333333333 |
| UbiCRE::V600E | 0.994 |C
n=3
n=5
25um
25um
Ki67
 UbiCRE UbiCRE::V600E
50um
50um
without tamoxifen
P=0.10 ns
### Chart
| Category | |
|---|---|
| UbiCRE | 0.9516666666666668 |
| UbiCRE::V600E | 0.5188 |D
n=3
n=5
25um
25um
p21CIP1
 UbiCRE UbiCRE::V600E
50um
50um
without tamoxifen
