# Supplementary material for: Early differential responses elicited by BRAFV600E in adult mouse models
Source: Cell Death Dis. 2022 Feb 10;13(2):142. doi: 10.1038/s41419-022-04597-z (PMC8831492; doi:10.1038/s41419-022-04597-z)
Supplement: Supplementary file 10 — Supplementary Figure 10 [file 41419_2022_4597_MOESM10_ESM.pptx]

## Slide 1
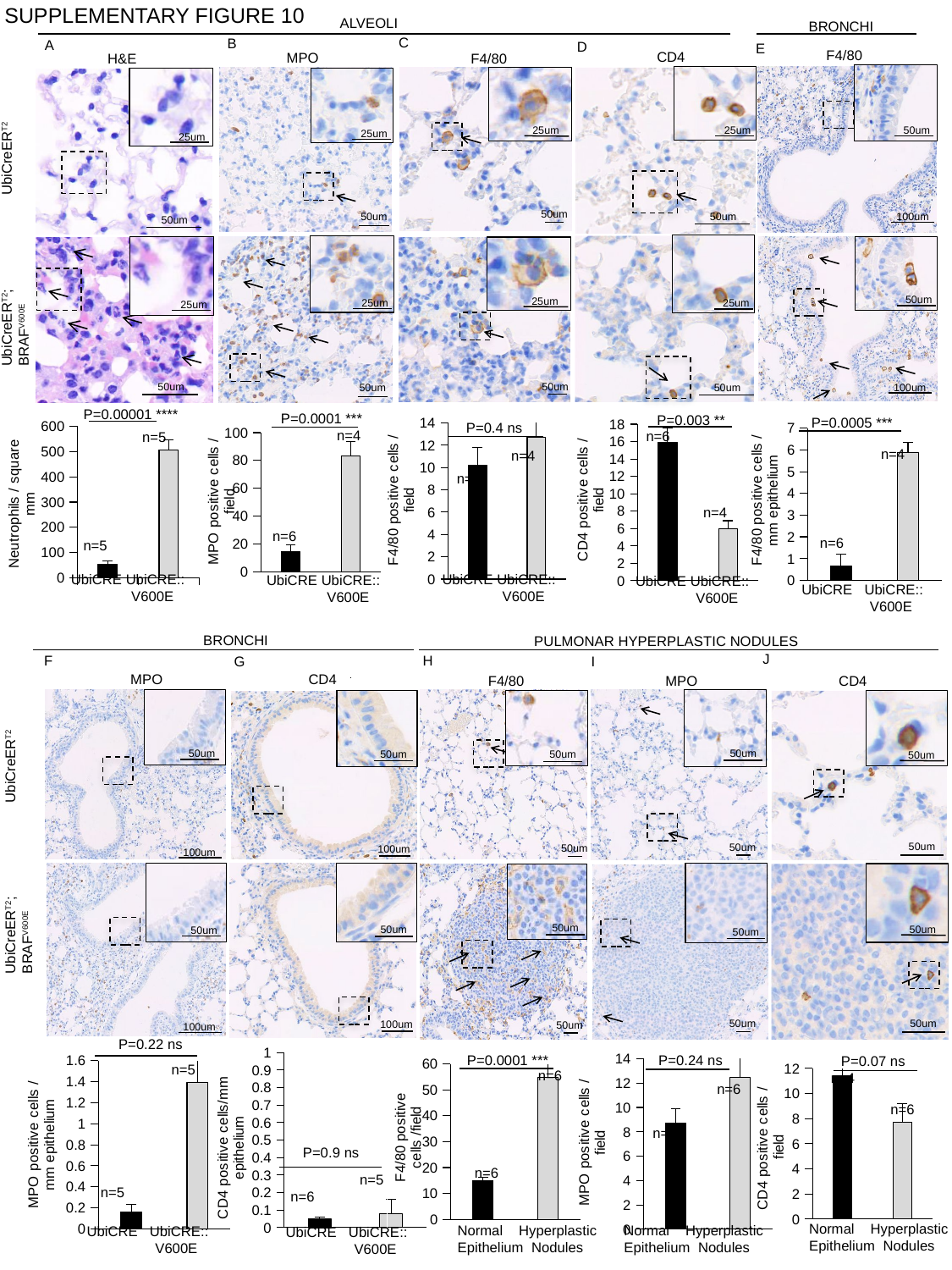

SUPPLEMENTARY FIGURE 10
ALVEOLI
BRONCHI
C
B
A
D
E
F4/80
CD4
MPO
F4/80
H&E
50um
25um
25um
25um
25um
UbiCreERT2
50um
50um
50um
100um
50um
50um
25um
25um
25um
25um
UbiCreERT2;
BRAFV600E
50um
50um
100um
50um
50um
P=0.00001 ****
P=0.0001 ***
P=0.003 **
P=0.0005 ***
### Chart
| Category | |
|---|---|
| UbiCre | 10.2 |
| UbiCre:V600E | 12.7 |
### Chart
| Category | |
|---|---|
| UbiCre | 14.3 |
| UbiCre::V600E | 83.4 |P=0.4 ns
### Chart
| Category | |
|---|---|
| UbiCre | 15.9 |
| UbiCre:V600E | 6.0 |
### Chart
| Category | |
|---|---|
| UbiCRE | 53.0 |
| UbiCRE::V600E | 507.0 |
### Chart
| Category | |
|---|---|
| UbiCRE | 0.6483333333333333 |
| UbiCRE::V600E | 5.8625 |n=4
n=6
n=5
n=4
n=4
n=5
n=4
n=6
n=6
n=5
 UbiCRE UbiCRE::
 V600E
 UbiCRE UbiCRE::
 V600E
 UbiCRE UbiCRE::
 V600E
 UbiCRE UbiCRE::
 V600E
UbiCRE UbiCRE::
 V600E
BRONCHI
PULMONAR HYPERPLASTIC NODULES
J
H
F
G
I
CD4
MPO
CD4
MPO
F4/80
UbiCreERT2
50um
50um
50um
50um
50um
50um
50um
50um
100um
100um
UbiCreERT2;
BRAFV600E
50um
50um
50um
50um
50um
50um
50um
50um
100um
50um
100um
P=0.22 ns
### Chart
| Category | |
|---|---|
| UbiCRE | 0.05 |
| UbiCRE::V600E | 0.08 |
### Chart
| Category | |
|---|---|
| Normal epithelium | 11.4 |
| Hyperplastic nodule | 7.7 |P=0.0001 ***
P=0.24 ns
P=0.07 ns
### Chart
| Category | |
|---|---|
| Normal epithelium | 8.7 |
| Hyperplastic nodule | 12.5 |
### Chart
| Category | |
|---|---|
| UbiCRE | 0.16 |
| UbiCRE::V600E | 1.39 |
### Chart
| Category | |
|---|---|
| Normal epithelium | 15.0 |
| Hyperplastic nodule | 54.8 |n=5
n=6
n=4
n=6
n=6
n=6
P=0.9 ns
n=6
n=5
n=5
n=6
 Normal Hyperplastic
 Epithelium Nodules
 Normal Hyperplastic
 Epithelium Nodules
 Normal Hyperplastic
 Epithelium Nodules
UbiCRE UbiCRE::
 V600E
UbiCRE UbiCRE::
 V600E
