# Supplementary material for: Early differential responses elicited by BRAFV600E in adult mouse models
Source: Cell Death Dis. 2022 Feb 10;13(2):142. doi: 10.1038/s41419-022-04597-z (PMC8831492; doi:10.1038/s41419-022-04597-z)
Supplement: Supplementary file 11 — Supplementary Figure 11 [file 41419_2022_4597_MOESM11_ESM.pptx]

## Slide 1
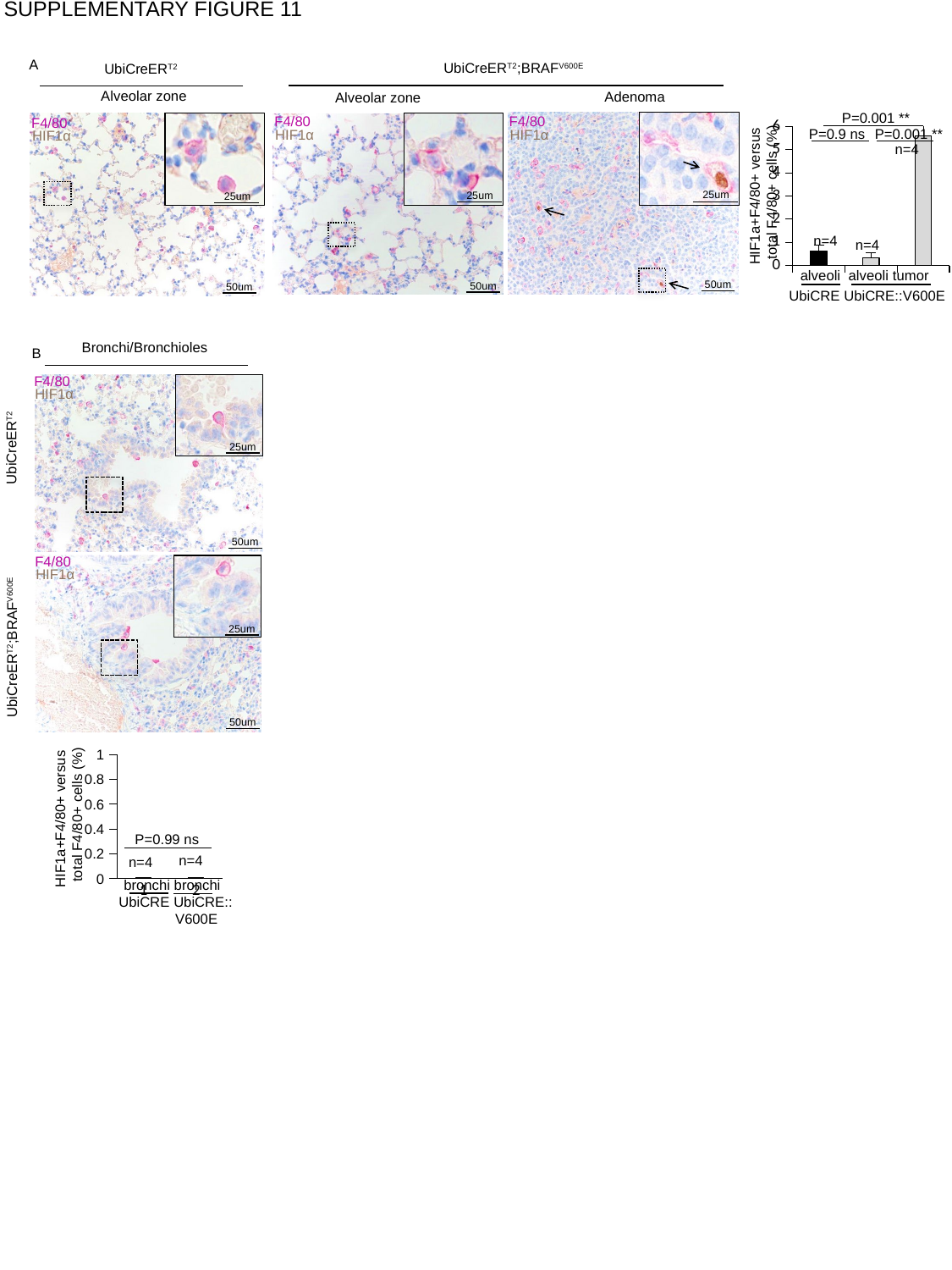

SUPPLEMENTARY FIGURE 11
A
UbiCreERT2;BRAFV600E
UbiCreERT2
Alveolar zone
Adenoma
Alveolar zone
P=0.001 **
F4/80
F4/80
F4/80
### Chart
| Category | |
|---|---|
| wt | 0.61 |
| mut | 0.34 |
| tum | 5.6 |P=0.9 ns
P=0.001 **
HIF1α
HIF1α
HIF1α
n=4
HIF1a+F4/80+ versus
 total F4/80+ cells (%)
25um
25um
25um
n=4
n=4
alveoli alveoli tumor
50um
50um
50um
 UbiCRE UbiCRE::V600E
Bronchi/Bronchioles
B
F4/80
HIF1α
UbiCreERT2
25um
50um
F4/80
HIF1α
25um
UbiCreERT2;BRAFV600E
50um
### Chart
| Category | |
|---|---|HIF1a+F4/80+ versus
 total F4/80+ cells (%)
P=0.99 ns
n=4
n=4
 bronchi bronchi
 UbiCRE UbiCRE::
 V600E
