# Supplementary material for: Early differential responses elicited by BRAFV600E in adult mouse models
Source: Cell Death Dis. 2022 Feb 10;13(2):142. doi: 10.1038/s41419-022-04597-z (PMC8831492; doi:10.1038/s41419-022-04597-z)
Supplement: Supplementary file 12 — Supplementary Figure Legends [file 41419_2022_4597_MOESM12_ESM.docx]

**Early differential responses elicited by BRAF^V600E^ in adult mouse models**

Giuseppe Bosso^1^, Pablo Lanuza-Gracia^1,2^, Sergio Piñeiro-Hermida^1,2^, Merve Yilmaz^1^, Rosa Serrano^1^ and Maria A. Blasco^1*^

**Short title: Rapid differential responses induced by BRAF^V600E^ *in vivo***

^1^ Telomeres and Telomerase Group, Molecular Oncology Program, Spanish National Cancer Centre (CNIO), Melchor Fernández Almagro 3, Madrid, E-28029, Spain.

^2^ These authors equally contributed to this work

* *Correspondence:* Maria A. Blasco

Spanish National Cancer Research Centre (CNIO)

3 Melchor Fernandez Almagro

Madrid E-28029, Spain

Tel.: +34.91.732.8031

Fax: +34.91.732.8028

Email: [mblasco@cnio.es](mailto:mblasco@cnio.es)

**Supplementary Figure 1. UbiCRE^RT2^ drives basal spontaneous activation of BRAF^V600E^ which results in mouse overall lifespan reduction and development of skin lesions. (A)** Survival curves of mice with the indicated genotype. n= animals per group. *** P<0.001. (Log Rank test).  **(B)** Some (13%) BRAF^V600E^ mice starting from 11-12 weeks of age develop papilloma-like lesions in the absence of tamoxifen treatment. Arrows point to such skin lesions. **(C)** PCR analysis from different tissues derived from BRAF^V600E^ and control mice in the presence or absence of tamoxifen administration to detect spontaneous rearrangement of BRAF^LSL_V600E^ allele. Recombination of the BRAF^V600E^ allele was detected in papilloma-like skin lesion of untreated 11 weeks old BRAF^V600E^ mice and in the skin of 7 weeks old BRAF^V600E^-treated mice, but not in the brain, which has been used as a negative control.

**Supplementary Figure 2. UbiCRE^RT2^ drives a ubiquitous activation of BRAF^V600E^ allele which is lethal in adult mice (A)** Schematic representation of mouse genotypes and experimental procedure employed in this study. 4HT= 4-hydroxy tamoxifen; **(B)** Survival curves of mice with the indicated genotype. n= animals per group. *** P<0.001. (Log Rank test).  **(C)** Necropsy of control (left) and BRAF^V600E^ mice (right). Note that the mutant BRAF mice showed hepatic steatosis (indicated by black arrow). **(D)** PCR analysis from different tissues derived from BRAF^V600E^ and control mice to detect rearrangement of BRAF^LSL_V600E^ allele upon 4HT administration. Recombination of the BRAF^V600E^ allele was detected in all the organs analyzed. Brain was used as a negative control as 4HT does not cross efficiently the blood brain barrier. li: liver, br: brain, sb: small bowel, th: thyroids, sk: skin, lu: lungs, lb: large bowel, pa: pancreas, sp: spleen. **(E-I)** Representative images and quantifications showing (E) Hematoxilin Eosin (H&E) staining, (F) phospho-ERK (ppERK), (G) Cleaved Caspase 3 (CC3), (H) p21^CIP1^ and (I) γH2AX immunostainings in thyroid sections from BRAF^V600E^ and control mice. Quantifications were performed on five different areas of the sections in a random way. Data are expressed as mean ± SEM; n= animals per group. * P<0.05; ** P<0.01; *** P<0.001, ns= not significant. (T-Student’s test unpaired). Arrows point to selected positive cells for the indicated marker. Insets: magnifications of areas inside dashed squares.

**Supplementary Figure 3. Ubiquitous expression of BRAF^V600E^ results in decrease of Ki67 expression in spleen.** Representative images (top) and quantifications (bottom) showing Ki67 staining in spleen sections from BRAF^V600E^ and control mice. Quantifications were performed on at least five different areas of the spleen sections in a random way. Data are expressed as mean ± SEM; n= animals per group. n= animals per group. * P<0.05; ** P<0.01; *** P<0.001, ns=not significant. (T Student’s test unpaired). Arrows point to selected positive cells for the indicated marker.

**Supplementary Figure 4.** **Ubiquitous expression of BRAF^V600E^ results in overall increase of protein levels of p53, p27^KIP1^, p21^CIP1^, p19^ARF^, p16^INK4a^, p15^INK4b^ and γH2AX markers in lungs.** Representative images (top) and quantifications (bottom) showing immunoblot experiments in lungs from BRAF^V600E^ and control mice. Data are expressed as mean ± SEM; n= animals per group. (T Student’s test unpaired). Asterisks point to non-specific bands.

**Supplementary Figure 5.** **Ubiquitous expression of BRAF^V600E^ does not result in telomere damage either in normal/hyperplastic alveolar epithelium or in hyperplastic nodules.** Representative images (top) and quantifications (bottom) showing immuno-FISH experiments in lungs sections from BRAF^V600E^ and control mice. Quantifications of telomere damage of alveolar epithelium in control mice compared with either (**A**) non-tumor alveolar epithelium or (**B**) lung adenomas of BRAF^V600E^ mice were performed by screening at least 400 cells for each condition. Data are expressed as mean ± SEM; n= animals per group. (T Student’s test unpaired). ns= not significant. Arrows point to examples of telomere-induced foci (TIF), positive for telomeric probe (red) and for the DNA damage marker 53BP1 (green).

**Supplementary Figure 6. Effects of BRAF^V600E^ expression on cell cycle and senescence markers in induced non-tumor alveolar parenchyma and lung adenomas.** Representative images (from the left) and quantifications (right) showing (A) p21^CIP1^, (B) pRb (phosphorylated at Ser807 and Ser811), (C) pSMAD3 (phosphorylated at Ser423/pSer425), and (D) pSTAT3 (phosphorylated at Tyr705) immunostainings in normal alveolar epithelium of control mice (left), non-tumor alveolar parenchyma (center) and adenomas (right) from lung sections of BRAF^V600E^ mice. Quantifications were performed on four different random areas of at least four hyperplastic nodules sections and four different random areas of uninduced alveolar parenchyma. Data are expressed as mean ± SEM; n represents respectively the number of animals per group in “alveoli” samples and the number of adenomas in the “tumor” samples. For each condition at least 4 mice were used. * P<0.05; ** P<0.01; *** P<0.001, ns=not significant. (ANOVA test with Tukey’s post-hoc correction). Arrows point to selected positive cells for the indicated marker. Insets: magnifications of areas inside dashed squares. In (C) and (D) tumors are delimited by dashed line.

**Supplementary Figure 7. BRAF^V600E^ induction does not results in the enrichment of nuclear pSMAD3 in ATII cells from uninduced, induced non-tumor alveolar parenchyma and lung adenomas.** Representative images (from the left) and quantifications (right) showing SPC+ cells staining positive for pSMAD3 double immunostainings in normal alveolar epithelium of control mice (left), non-tumor areas (center) and adenomas (right) from lung sections of BRAF^V600E^ mice. Quantifications were performed on four to ten different random areas of at least four hyperplastic nodules sections and four to ten different random areas of uninduced alveolar parenchyma. Data are expressed as mean ± SEM; n represents respectively the number of animals per group in “alveoli” samples and the number of adenomas in the “tumor” samples. For each condition at least 4 mice were used. * P<0.05; ** P<0.01; *** P<0.001, ns=not significant. (ANOVA test with Tukey’s post-hoc correction). Arrows point to selected positive cells for the indicated marker.

**Supplementary Figure 8.** **BRAF^V600E^-induced transdifferentiating Club cells show decreased expression of CC10 but not p21^CIP1^, γH2AX, Ki67, pRb markers.**

**Ubiquitous expression of results in a Club cells transdifferentiation.** (**A**) Quantification of the intensity of CC10 marker in lung sections from BRAF^V600E^ and control mice. (**B-E**) Quantification of the percentage of (B) p21^CIP1^, (C) γH2AX, (D) Ki67, (E) pRb positive bronchial/bronchiolar cells which also stain positive or negative for SPC marker. Quantifications were performed on at least five different areas of the bronchi/bronchioles in a random way. Data are expressed as mean ± SEM; n= animals per group, * P<0.05; ns=not significant (T Student’s test unpaired). AU= Arbitrary Units.

**Supplementary Figure 9. Spontaneous activation of BRAF^V600E^ results in lung adenomas development but does not affect p21^CIP1^, p53, Ki67 expression either in non-tumor alveolar parenchyma or bronchial/bronchiolar epithelium.**

Representative images (left) and quantifications (right) showing (**A**) p21^CIP1^ and (**B**) p53 immunostainings in alveolar parenchyma and adenomas from BRAF^V600E^ and control mice in the absence of tamoxifen treatment. Representative images (left) and quantifications (right) showing bronchial/bronchiolar epithelial cells staining positive for (**C**) Ki67 and (**D**) p21^CIP1^ immunostainings in lung sections from BRAF^V600E^ and control mice. Quantifications were performed on five different areas of the sections in a random way. Data are expressed as mean ± SEM; n represents respectively the number of animals per group in “alveoli” samples as well as in C and D, and the number of adenomas in the “tumor” samples of A and B. * P<0.05; ** P<0.01; *** P<0.001, ns=not significant. (ANOVA test with Tukey’s post-hoc correction for A and B; T Student’s test unpaired for C and D). Arrows point to selected positive cells for the indicated marker. Insets: magnifications of areas inside dashed squares.

**Supplementary Figure 10. Ubiquitous expression of the oncogenic variant of BRAF results in neutrophils infiltration in alveoli and F4/80-positive cells infiltration in bronchial epithelium as well as in hyperplastic nodules.** Representative images (top) and quantifications (bottom) showing H/E, Myeloperoxidase (MPO), F4/80, and CD4 immunostainings in alveoli respectively (**A**), (**B**), (**C**), (**D**), in bronchi respectively (**E**) F4/80, (**F**) MPO and (**G**) CD4 and in hyperplastic nodules, respectively (**H**) F4/80, (**I**) MPO and (**J**) CD4 from BRAF^V600E^ and control mice. Quantifications in alveoli and bronchi were performed on at least five different areas of the lung sections in a random way. Quantifications in hyperplastic nodules were performed on four different random areas of at least four hyperplastic nodules sections in at least four BRAF^V600E^ mice and four different random areas of normal epithelium from at least 4 control mice. Data are expressed as mean ± SEM; n= animals per group, except for (H),(I) and (J) where n indicates the number of hyperplastic nodules. Data are expressed as mean ± SEM; n= animals per group. * P<0.05; ** P<0.01; *** P<0.001, ns=not significant. (T Student’s test unpaired). Arrows point to selected positive cells for the indicated marker. Insets: magnifications of areas inside dashed squares.

**Supplementary Figure 11. BRAF^V600E^ induction results in M1-like macrophages increase in lung adenomas only.** (**A**) Representative images (on the left) and quantifications (on the right) showing F4/80+ cells staining positive for HIF1α immunostainings in alveolar parenchyma and adenoma sections from BRAF^V600E^ and control mice. (**B**) Representative images (top) and quantifications (bottom) showing F4/80+ cells staining positive for HIF1α double immunostainings in bronchi/bronchiolar parenchyma of lung sections from BRAF^V600E^ and control mice. Quantifications were performed on at least five different areas of the lung sections in a random way. Data are expressed as mean ± SEM; n= animals per group. * P<0.05; ** P<0.01; *** P<0.001, ns=not significant. (ANOVA test with Tukey’s post-hoc correction (A), T Student’s test unpaired (B). Arrows point to selected positive cells for the indicated marker. Insets: magnifications of areas inside dashed squares.
