# Supplementary material for: Early differential responses elicited by BRAFV600E in adult mouse models
Source: Cell Death Dis. 2022 Feb 10;13(2):142. doi: 10.1038/s41419-022-04597-z (PMC8831492; doi:10.1038/s41419-022-04597-z)
Supplement: Supplementary file 13 — Materials and Methods [file 41419_2022_4597_MOESM13_ESM.docx]

**Early differential responses elicited by BRAF^V600E^ in adult mouse models**

Giuseppe Bosso^1^, Pablo Lanuza-Gracia^1,2^, Sergio Piñeiro-Hermida^1,2^, Merve Yilmaz^1^, Rosa Serrano^1^ and Maria A. Blasco^1*^

**Short title: Rapid differential responses induced by BRAF^V600E^ *in vivo***

^1^ Telomeres and Telomerase Group, Molecular Oncology Program, Spanish National Cancer Centre (CNIO), Melchor Fernández Almagro 3, Madrid, E-28029, Spain.

^2^ These authors equally contributed to this work

* *Correspondence:* Maria A. Blasco

Spanish National Cancer Research Centre (CNIO)

3 Melchor Fernandez Almagro

Madrid E-28029, Spain

Tel.: +34.91.732.8031

Fax: +34.91.732.8028

Email: [mblasco@cnio.es](mailto:mblasco@cnio.es)

**Materials and Methods**

**Murine models**

BRAF^LSLV600E^ mice were described previously(1–3). This mouse model was crossed with a mouse strain carrying ubiquitously expressed, tamoxifen-activated recombinase, UBC-CreER^T2^ (4), to generate UBC-CreER^T2/+^;BRAF^LSL_V600E/+^ mice. These mice received intraperitoneal injections of 4-hydroxy tamoxifen (Sigma H6278) (1mg/injection, 3-4 injections, 1 injection per day for 3 or 4 consecutive days). Mat

All mice were maintained at the Spanish National Cancer Research Centre under specific pathogen-free conditions in accordance with the recommendations of the Federation of European Laboratory Animal Science Associations (FELASA). All animal experiments were approved by our Institutional Animal Care and Use Committee (IACUC) and by the Ethical Committee for animal experimentation (CEIyBA) (PROEX 106.7/20). We followed the Reporting in Vivo Experiments (ARRIVE) guidelines developed by the National Centre for the Replacement, Refinement & Reduction of Animals in Research (NC3Rs). Both male and female mice, with mixed background, were used for the experiments.

**Immuno-FISH**

Immuno-FISH was performed in formalin-ﬁxed parafﬁn-embedded mouse lung sections to identify telomeric induced foci (TIF) as previously described(5,6). Immuno-FISH was performed as follows: after deparaffination and citrate antigen retrieval, samples were permeabilized for 3 h in PBS1X-0.5% Triton, blocked for 2h with 10% fetal bovine serum and 1h with 5% BSA in PBS1X-0.1%Triton-10mM Glycine (PBSTG), and immunoﬂuorescence with anti-53BP1 rabbit antibody (Novus Biologicals NB100-304) diluted 1:500 was performed. Samples were incubated O/N at 4 °C with the primary antibody in PBSTG. Slides were further washed with PBSTG and incubated with 488-Alexa labeled secondary antibody in DAKO antibody diluent reagent (S3022). After immunoﬂuorescence, samples were ﬁxed for 20 min in 4% paraformaldehyde in PBS1X and followed by FISH. Brieﬂy, samples were washed with PBS and dehydrated in Ethanol 70, 90 and 100%. The samples were then incubated with a telomeric PNA probe labeled with CY3 (Panagene) in 50% formamide for 30 min, washed in the presence of 50% formamide and counterstained with DAPI. TIF were identiﬁed by colocalization of CY3 and 488-Alexa double positive spots. Confocal microscopy was performed at room temperature with a laser-scanning microscope (TCS SP5; Leica) using a Plan Apo 63Å-1.40 NA oil immersion objective (HCX; Leica). Maximal projection of Z-stack images generated using advanced ﬂuorescence software (LAS) was analyzed with the Deﬁniens XD software package. The DAPI images were used to detect signals inside the nuclei.

**Immunohistochemistry Analyses in Tissue Sections**

Tissues were fixed in 10% buffered formalin, embedded in paraffin wax and sectioned at 5 mm. For histological examination sections were stained with hematoxylin and eosin, according to standard procedures as previously described(7). CC3 Cleaved Caspase 3 Asp175 (Cell Signaling Technology 9661), CC10 (Santa Cruz Biotechnology sc-9772), CD4 (Cell Signaling Technology 25229, prosurfactant protein C (millipore AB3786), p21 (291 H/B5, homemade), γH2AX Ser 139 (Millipore 05-636), PPERK Thr202/Tyr204 (Cell Signaling Tehcnology 9378), Ki67 (Cell Signaling 12202), MPO (Dako A0398), F4-80 (ABD Serotec MCA497), p16 (33B, homemade), p19 ARF (sc-32748 Santa Cruz), pRb (ser807/811, #9308, Cell Signaling), pSMAD3 (ser423/425 ab52903, Abcam), pSTAT3 (tyr705, #9145 Cell Signaling), p53 (POE316A, homemade), cyclin D1 (M3635, Dako), c-MYC (ab32072, Abcam), PPARγ (Cell Signaling, #2435), HIF1α (Cell Signaling, #36169), 8-hydroxy-2’-deoxyguanosine (Abcam, ab48508), 4-hydroxy-2-nonenal (Alpha Diagnostic, HNE11-S) antibodies were used for immunohistochemistry in tissue sections. Pictures were taken using Olympus AX70 microscope. The percentage of positive cells was identified by eye and the areas were calculated by ImageJ and Zen 3.1 (Zeiss) softwares.

**Protein extract preparation and Western Blot**

Protein extracts were obtained as follows: 45mg of lung for each mouse were mechanically homogenized in 850ul lysis buffer (50mM TrisHCl pH 7.5, 420mM NaCl, 1% Triton, 1mM EDTA, 2.5mM MgCl2, protease inhibitors) in *BERTIN Precellys 24 Lysis & Homogenization* machine, incubated 30 min on ice in agitation, sonicated 10 seconds, centrifuged at 14000g for 20min at 4ºC. The recovered supernatant was passed through a 0.22 filter, aliquoted, flash-frozen in liquid nitrogen and stored at -80ºC. Protein concentration was determined using the Bio‐Rad DC Protein Assay (Bio‐Rad). 40µg of nuclear protein extracts were separated in SDS–polyacrylamide gels by electrophoresis. After protein transfer onto nitrocellulose membrane, the membranes were incubated with the indicated antibodies: monoclonal anti-actin 1:5000 (A5441, Sigma), anti-BRAF 1:200 (F-7, sc-5284, Santa Cruz), anti-BRAF^V600E^ 1:300 (31-1042-00 RevMAB Biosciences USA), anti-γH2AX Ser139 1:5000 (Merck 05-636), homemade rat anti-p15^INK4b^ clone PAT65B (neat supernatant), homemade rat anti-p16^INK4a^ clone PABLO33B (neat supernatant), handmade rat anti-p19^ARF^ clone PIL346C (neat supernatant), homemade rat anti-p27^KIP1^ clone SON82D (neat supernatant), homemade rat anti-p21^CIP1^ clone HUGO291 (neat supernatant), homemade rat anti-p53 clone POE316A (neat supernatant). Antibody binding was detected after incubation with a secondary antibody coupled to horseradish peroxidase using chemiluminescence with ECL detection KIT (GE Healthcare) with Chemidoc (Biorad). For the quantification, protein‐band intensities were quantified by densitometric analysis with ImageLab software (Biorad). The total levels of each protein analyzed have been normalized versus actin and the mean of the specific protein/actin ratio deriving from at least 3 different replicates has been used to generate the chart as previously described(8).

**PCR**

DNA of tissue samples was extracted using Phenol:Chloroform:Isoamyl:Alcohol (Sigma). We determined Cre-mediated recombination by using the following PCR program: 94°C for 3min, followed by 33 cycles of 94°C denaturation for 25 sec, 25 sec annealing at 55°C, elongation at 73°C for 45 sec, followed by a 4min 73°C elongation step with the following primes: Fw 5’-TGAGTATTTTTGTGGCAACTGC and Rev 5’-CTCTGCTGGGAAAGCGGC. This oligonucleotide primer pair hybridizes in intron 14 flanking the cassette insertion site. These conditions produce diagnostic PCR products of 185bp for the wild-type BRAF and 308bp for BRAF^V600E^alleles and a 335bp PCR product for the Cre-activated BRAF^V600E^ allele. The samples were resolved in a 3% agarose gel.

**Quantification and statistical analysis**

Immunohistochemistry quantifications were performed by direct cell counting by using Zen3.1 Zeiss and Image J softwares. ImmunoFISH quantifications were carried out by direct counting of cells and 53BP1 foci on single plans of each z-stack by using LAS X software (Leica). Unpaired Student's t test (two-tailed), ANOVA followed by Tukey’s post-hoc correction, Log Rank test were used to determine statistical significance. P values of less than 0.05 were considered significant. *p<0.05, **p<0.01, ***p<0.001. Statistical analysis was performed using Microsoft® Excel 2016 and GraphPad/PRISM8. For animal studies no blinding/randomization was done/used. The number of mice per each experiment as well as the size of the experiments were obtained by performing power analysis.

**Data Availability**

The datasets and other information that support the findings of this study are available from the corresponding author upon reasonable request.
