# Supplementary material for: Early differential responses elicited by BRAFV600E in adult mouse models
Source: Cell Death Dis. 2022 Feb 10;13(2):142. doi: 10.1038/s41419-022-04597-z (PMC8831492; doi:10.1038/s41419-022-04597-z)
Supplement: Supplementary file 14 — Agreements and responses from co-authors with change [file 41419_2022_4597_MOESM14_ESM.docx]

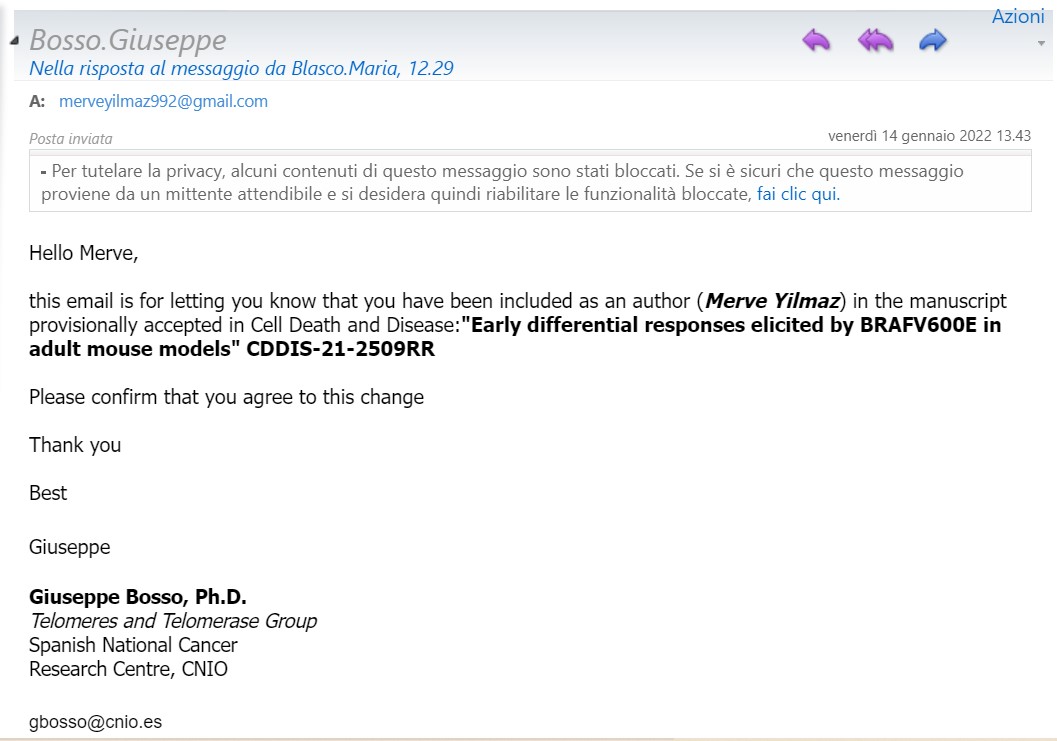

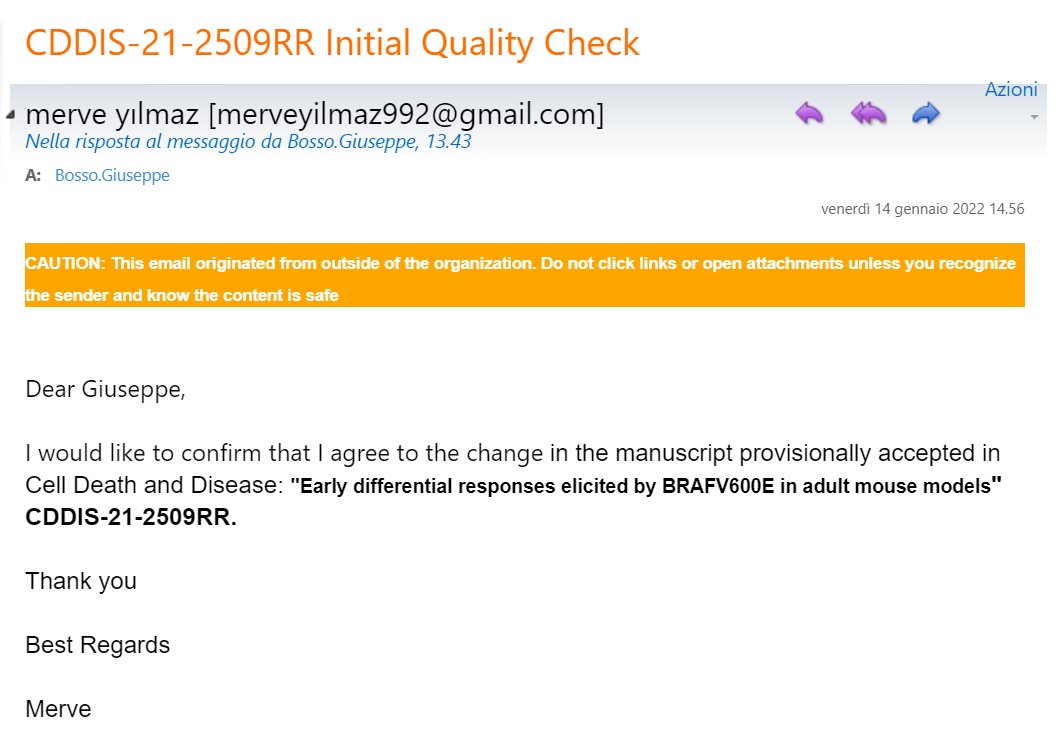


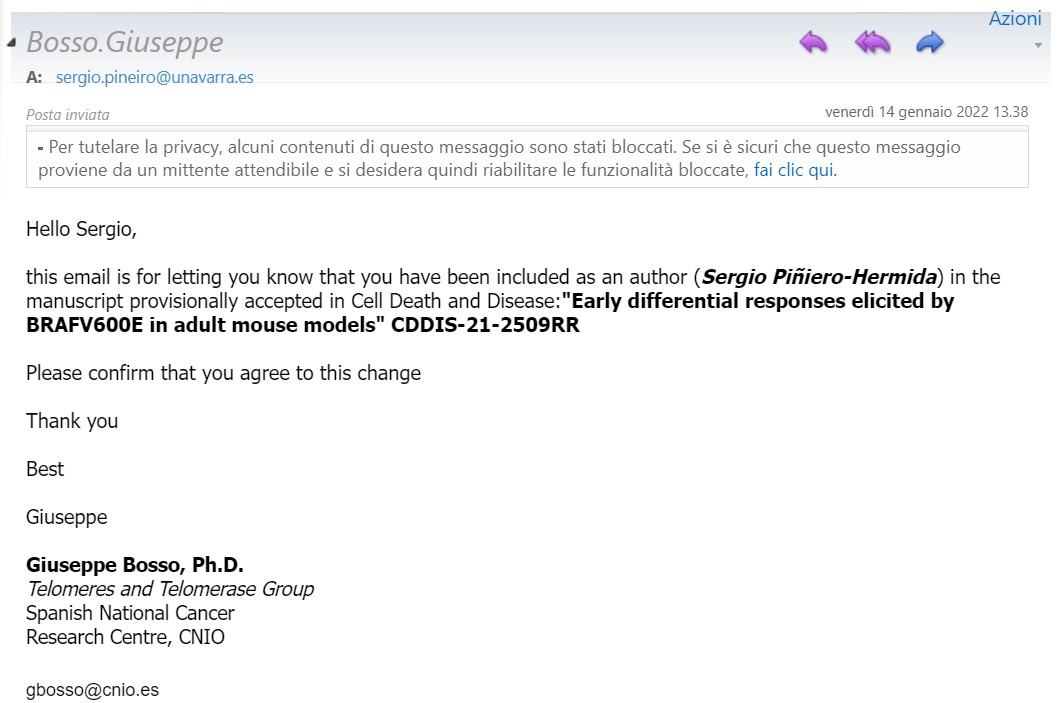

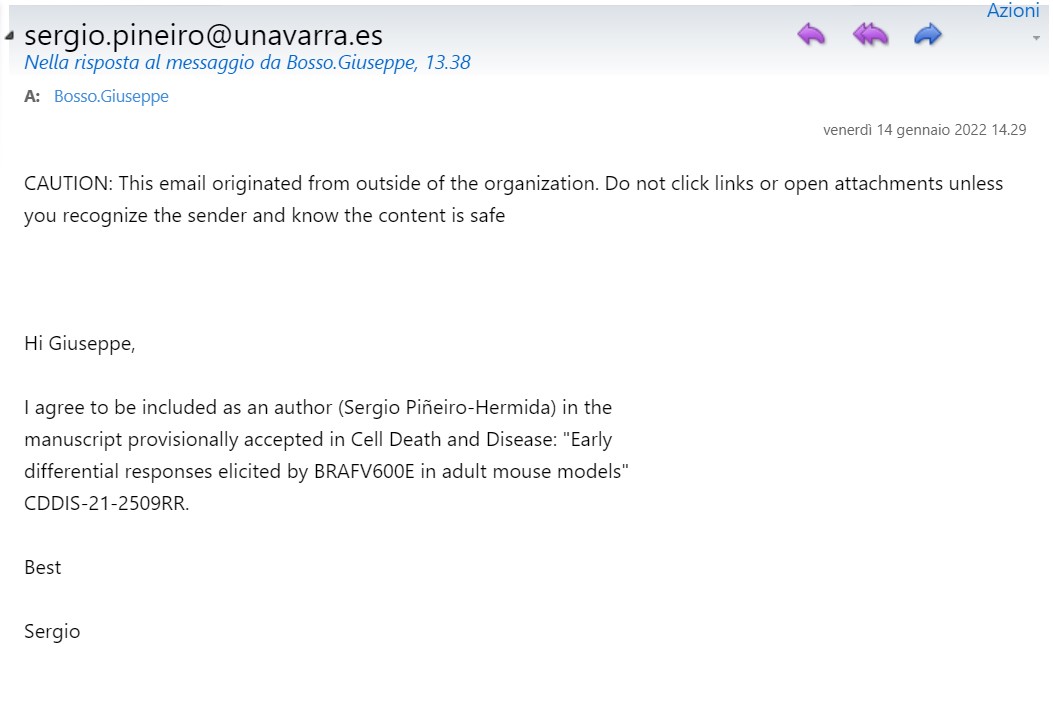


CDDIS-21-2509RR Initial Quality Check

sergio.pineiro@unavarra.es



Azioni



*Nella risposta al messaggio da Bosso.Giuseppe, 13.38*

**A:**

 Bosso.Giuseppe

venerdì 14 gennaio 2022 14.29

CAUTION: This email originated from outside of the organization. Do not click links or open attachments unless you recognize the sender and know the content is safe


Hi Giuseppe,

I agree to be included as an author (Sergio Piñeiro-Hermida) in the
manuscript provisionally accepted in Cell Death and Disease: "Early
differential responses elicited by BRAFV600E in adult mouse models"
CDDIS-21-2509RR.

Best

Sergio


El Vie, 14 de Enero de 2022, 13:38, Bosso.Giuseppe escribió:
> Hello Sergio,
>
> this email is for letting you know that you have been included as an
> author (Sergio Piñiero-Hermida) in the manuscript provisionally accepted
> in Cell Death and Disease:"Early differential responses elicited by
> BRAFV600E in adult mouse models" CDDIS-21-2509RR
>
> Please confirm that you agree to this change
>
> Thank you
>
> Best
>
> Giuseppe
>
> Giuseppe Bosso, Ph.D.
> Telomeres and Telomerase Group
> Spanish National Cancer
> Research Centre, CNIO
>
> gbosso@cnio.es
> Phone +34 91 732 8000 (ext. 3411)
>
> Melchor Fernández Almagro, 3
> 28029 Madrid, Spain
> www.cnio.es<[https://www.cnio.es/](https://mail.cnio.es/owa/redir.aspx?C=OXOpCHntZ3uhU-QmZzE3GkYq7xRLL-TL-IEmTDLndVASIrjxbdfZCA..&URL=https%3a%2f%2fwww.cnio.es%2f)>
>
> [CNIO stop cancer. Excelencia Severo Ochoa 2012-2019]

CDDIS-21-2509RR Initial Quality Check




merve yılmaz [merveyilmaz992@gmail.com]



venerdì 14 gennaio 2022 14.56

**CAUTION: This email originated from outside of the organization. Do not click links or open attachments unless you recognize the sender and know the content is safe**

Dear Giuseppe,

I would like to confirm that I agree to the change in the manuscript provisionally accepted in Cell Death and Disease: **"Early differential responses elicited by BRAFV600E in adult mouse models" CDDIS-21-2509RR.**

Thank you

Best Regards

Merve



*Bosso.Giuseppe*



Azioni



*Nella risposta al messaggio da Blasco.Maria, 12.29*

**A:**

 merveyilmaz992@gmail.com

*Posta inviata*

venerdì 14 gennaio 2022 13.43



Per tutelare la privacy, alcuni contenuti di questo messaggio sono stati bloccati. Se si è sicuri che questo messaggio proviene da un mittente attendibile e si desidera quindi riabilitare le funzionalità bloccate, [fai clic qui.](https://mail.cnio.es/owa/)

Hello Merve,

this email is for letting you know that you have been included as an author (***Merve Yilmaz***) in the manuscript provisionally accepted in Cell Death and Disease:**"Early differential responses elicited by BRAFV600E in adult mouse models" CDDIS-21-2509RR**

Please confirm that you agree to this change

Thank you

Best

Giuseppe

**Giuseppe Bosso, Ph.D.**

*Telomeres and Telomerase Group*

Spanish National Cancer

Research Centre, CNIO

gbosso@cnio.es
Phone +34 91 732 8000 (ext. 3411)

Melchor Fernández Almagro, 3
28029 Madrid, Spain
[www.cnio.es](https://mail.cnio.es/owa/redir.aspx?C=i8H0lqNoHDwKSvyOPIVgfG3ZqfRGDak5GgwYxkAQr507i1EbbtfZCA..&URL=https%3a%2f%2fwww.cnio.es%2f)
